# A high-energy Laue X-ray emission spectrometer at the FXE instrument at the European XFEL

**DOI:** 10.1107/S1600577525001389

**Published:** 2025-03-31

**Authors:** X. Huang, Y. Uemura, F. Ardana-Lamas, P. Frankenberger, M. Knoll, H. Yousef, H. Wang, S. Heder, M. Nachtegaal, G. Smolentsev, L. Wang, L. F. Zhu, C. Milne, F.A. Lima

**Affiliations:** a European XFEL Holzkoppel 4 22869Schenefeld Germany; b Center for Energy and Environmental Sciences Paul Scherrer Institute CH-5232Villigen Switzerland; c Center for Photon Science Paul Scherrer Institute CH-5232Villigen Switzerland; d Department of Modern Physics University of Science and Technology of China 230026Hefei People’s Republic of China; ESRF – The European Synchrotron, France

**Keywords:** X-ray free-electron lasers, X-ray spectrometers, X-ray emission spectroscopy, DuMond geometry, ultrafast science, Laue crystal analyzers

## Abstract

The high-energy-resolution X-ray emission spectroscopy (XES) spectrometers available at the Femtosecond X-ray Experiment (FXE) instrument of the European XFEL operate in Bragg (reflective) geometry, with optimum performance in the range between 5 and 15 keV. However, they quickly lose efficiency above around 15 keV due to the decrease in reflectivity of the crystal analyzers at such high photon energies. This hampers high-energy-resolution spectroscopy experiments on heavy elements (*e.g.* 4*d* metals), which thus do not fully profit from the high-photon-energy capabilities of the European XFEL. Here we present the design, implementation and performance of a novel high-resolution XES spectrometer operating in Laue (transmission) geometry optimized for measurements at high photon energies (>15 keV). The High-Energy Laue X-ray emIssiOn Spectrometer (HELIOS) operates mainly in dispersive mode by placing the crystal analyzer inside or outside the Rowland circle. The Laue spectrometer performance in terms of energy resolution and efficiency is presented and discussed. Two Laue analyzers, silicon and quartz, have been tested at SuperXAS of the Swiss Light Source and at FXE of the European XFEL. The quartz analyzer was found to be about 2.7 times more efficient than the silicon one. The Laue spectrometer energy resolution (Δ*E*/*E*) reached at the FXE instrument is around 1.2 × 10^−4^. Depending on different user requests, the resolution can be further increased by using higher diffraction orders. The new Laue spectrometer increases the existing portfolio of XES spectrometers at FXE, enabling efficient implementation of ultrafast X-ray spectroscopies with high energy resolution at photon energies above 15 keV. This spectrometer will allow the expansion of studies in the field of ultrafast sciences, particularly including investigation of 4*d* elements using hard X-rays.

## Abstract 2

A new X-ray spectrometer for high photon energies based on Laue analyzer crystals is presented. Its performance in terms of energy resolution and efficiency is discussed. Niobium *K*α and *K*β emission data collected with this Laue spectrometer are given.

## 1. Introduction

The European X-ray Free Electron Laser (XFEL) facility provides unique scientific capabilities by generating ultrashort X-ray pulses at MHz repetition rates and, in particular, high X-ray photon energies (>15 keV) covering the *K* edges of 4*d* elements and *L* edges of 5*f* elements (Chen *et al.*, 2021 ▸). Accessing those edges results in distinctive opportunities for applying X-ray core level spectroscopies in the investigation of the temporal evolution of physical, chemical and biological processes in condensed matter. X-ray spectroscopies have the advantage of being sensitive to the electronic configurations and local structures around the absorbing element, and have routinely been employed in the study of photo-excited processes of materials at synchrotron radiation and XFEL facilities (Milne *et al.*, 2014 ▸; Gawelda *et al.*, 2016 ▸; de Groot *et al.*, 2024 ▸; Bergmann *et al.*, 2021 ▸). To date, the majority of ultrafast spectroscopic experiments conducted on free-electron lasers in the hard X-ray regime have been limited to ∼4.5–10 keV, with time-resolved (t.r.) X-ray absorption and emission (XAS and XES, respectively) being well established and broadly used for systems containing 3*d* (*K* edge) and 5*d* (*L* edge) metals (Zhang *et al.*, 2014 ▸; Canton *et al.*, 2015 ▸; Levantino *et al.*, 2015 ▸; Mara *et al.*, 2017 ▸; Lemke *et al.*, 2017 ▸; Katayama *et al.*, 2019 ▸; Kunnus *et al.*, 2020 ▸; Smolentsev *et al.*, 2020 ▸; Bacellar *et al.*, 2023 ▸; Sension *et al.*, 2023 ▸; Nowakowski *et al.*, 2024 ▸; Naumova *et al.*, 2024 ▸). Ultrafast t.r. X-ray spectroscopies have rarely been employed in very hard X-ray regimes, mainly due to the unavailability of free-electron laser sources producing sufficiently intense pulses above 15 keV and efficient high-resolution X-ray spectrometers for such experiments. In fact, high-resolution spectrometers optimized for high photon energies are rare even on synchrotron sources (Ravel *et al.*, 2018 ▸; Jagodziński *et al.*, 2019 ▸).

Static high-energy-resolution X-ray spectroscopies on systems containing 3*d* (*K* edges) and 5*d* (*L* edges) metals are becoming routine and are used to address a variety of relevant science questions (Lancaster *et al.*, 2011 ▸; Sá *et al.*, 2014 ▸; Torres Deluigi *et al.*, 2014 ▸; Pollock & DeBeer, 2015 ▸; Hunault *et al.*, 2017 ▸; Schuth *et al.*, 2018 ▸; Castillo *et al.*, 2021 ▸; Cutsail & DeBeer, 2022 ▸). On the other hand, only recently have high-resolution XAS and XES on the *K* edge of 4*d* metals started to become more common, indicating the importance of expanding the capabilities for performing such experiments with improved efficiency (Doonan *et al.*, 2005 ▸; Lima *et al.*, 2013 ▸; Bjornsson *et al.*, 2014 ▸; Lezcano–González *et al.*, 2016 ▸; Ravel *et al.*, 2018 ▸; Castillo *et al.*, 2020 ▸). In this sense, extending the high-energy-resolution X-ray spectroscopy capabilities at XFELs to high photon energies is a natural development. This would significantly expand the scope of science covered, allowing the investigation of photo-induced processes in materials containing 4*d* elements at XFELs, *e.g.* charge-transfer dynamics on dyads containing 4*d* metals (Ducrot *et al.*, 2016 ▸; Huijser *et al.*, 2018 ▸), the photocatalytic properties of niobium and molybdenum nanoparticles (Su *et al.*, 2021 ▸; Ducrot *et al.*, 2016 ▸) and ruthenium dye-sensitized nanoparticles for solar cell applications (Zhang *et al.*, 2011 ▸), the ultrafast evolution of strongly correlated materials (Witczak-Krempa *et al.*, 2014 ▸) and new candidates for battery cathodes and anodes (Han *et al.*, 2023 ▸), among others. These materials often exhibit unique competition between fundamental interactions. For instance, the dominant role of electronic correlations and spin–orbit coupling may be inverted in 3*d* and 4*d* metals, resulting in distinct physical behaviors on materials containing 4*d* metals compared with their 3*d* counterparts (Cao & Delong, 2013 ▸). Moreover, spectroscopic studies at such high energies would nicely match the advantages of scattering and diffraction experiments, potentially done in parallel in this regime, in terms of wider coverage of the momentum transfer (Dunne *et al.*, 2023 ▸). Additionally, core-level spectroscopy in a high-photon-energy regime suffers from an intrinsic limitation in the energy resolution due to the large core-hole lifetime broadening (Ogasawara *et al.*, 1994 ▸). This is another motivation for developing high-energy-resolution spectrometers (Hiraoka *et al.*, 2013 ▸) to circumvent this limitation by resonant XES and high-energy-resolution fluorescence-detected XAS (HERFD-XAS) (Lima *et al.*, 2013 ▸; Hämäläinen *et al.*, 1991 ▸).

Currently, there are two X-ray emission spectrometers installed at the Femtosecond X-ray Experiment (FXE) instrument which have been successfully used in several user experiments since inauguration (Naumova *et al.*, 2020*a* ▸; Naumova *et al.*, 2020*b* ▸; Kinschel *et al.*, 2020 ▸; Bacellar *et al.*, 2020 ▸; Bacellar *et al.*, 2023 ▸; Sension *et al.*, 2023 ▸; Canton *et al.*, 2023 ▸; Naumova *et al.*, 2024 ▸; Nowakowski *et al.*, 2024 ▸; Sension *et al.*, 2024 ▸). Both von Hamos and Johann X-ray spectrometers, previously described by Galler *et al.* (2019 ▸) and Lima *et al.* (2023 ▸), operate in reflective Bragg diffraction geometry using silicon and germanium crystal analyzers. They typically provide an energy resolution on the order of 1 eV or even below. However, these Bragg-reflection analyzers present a significant limitation in the overall spectrometer efficiency at high photon energies. The X-ray penetration depth on the Si and Ge analyzers becomes significant at energies above ∼15 keV, with a consequent quick decrease in the crystal reflectivity as the energy increases. Even though using higher-index diffraction orders can result in an enhancement in the crystal reflectivity to partially compensate this, the corresponding Darwin width gets remarkably narrow, balancing the gain in reflectivity and preventing a reasonable efficiency increase (Shvyd’Ko, 2004 ▸; Szlachetko *et al.*, 2013 ▸; Jagodziński *et al.*, 2019 ▸). Hence, the applicability of those spectrometers in investigations of heavier elements, for example 4*d* metals, is hampered (Castillo *et al.*, 2020 ▸). This not only represents a significant restriction on the science cases that can be tackled at FXE but also a limit on the exploration of the ultrafast high X-ray photon energy capabilities of the European XFEL.

In order to overcome these limitations and increase the existing portfolio of high-resolution X-ray spectrometers at FXE, we have designed a dedicated high-energy Laue (transmission-type) XES spectrometer which was recently installed at FXE. The Laue-transmission geometry (Hiraoka *et al.*, 2013 ▸; Szlachetko *et al.*, 2013 ▸; Ravel *et al.*, 2018 ▸; Jagodziński *et al.*, 2019 ▸), in which the diffracted beam is transmitted through a thin crystal analyzer, results in higher efficiency and comparable energy resolution with that reached using Bragg-reflection geometry. Noteworthy, in Laue geometry the crystal planes are in the same direction as the beam, rather than perpendicular. Herein we present our High-Energy Laue X-ray emIssiOn Spectrometer (HELIOS) with an optimized analyzer design for improved efficiency. An energy resolution (Δ*E*/*E*) of ∼1.2 × 10^−4^ was reached and the improved efficiency is demonstrated by comparing the XES signal magnitude obtained with the Laue spectrometer with that measured simultaneously with the FXE von Hamos spectrometer and also with the Laue-type spectrometer reported in the work of Jagodziński *et al.* (2019 ▸). Additionally, the Laue spectrometer reported here provides a less distorted emission image when operating in dispersive mode compared with the Laue spectrometer reported in the work of Ravel *et al.* (2018 ▸). This is particularly relevant for pump–probe experiments in which consecutive laser-on and laser-off spectra are subtracted and any artifacts arising from image processing should be minimized. The successful inauguration of this new emission spectrometer, the only such equipment at XFELs, should allow major questions in the field of ultrafast science to be addressed.

## 2. Spectrometer design concepts and optimization

Laue-type analyzers have been used for more than half a century in several spectroscopic applications, including identification of fluorescence lines of very high *Z* atoms and ions (Borchert *et al.*, 1975 ▸; Widmann *et al.*, 1997 ▸), measurements of broad Compton profiles with high energy resolution (Suortti *et al.*, 1999 ▸; Itou *et al.*, 2001 ▸; Hiraoka *et al.*, 2001 ▸; Hiraoka *et al.*, 2005 ▸) and to resolve fluorescence lines and suppress background scattering for XAS detection (Zhong *et al.*, 1999 ▸; Kropf *et al.*, 2003 ▸; Kropf *et al.*, 2005 ▸; Wakisaka *et al.*, 2017 ▸). They have also been used in an X-ray Raman spectrometer utilizing ∼20 keV photons (Hiraoka *et al.*, 2013 ▸). However, the energy resolution achieved in these experiments was usually limited to Δ*E*/*E* ≃ 10^−3^, with the work from Hiraoka *et al.* reporting Δ*E*/*E* ≃ 10^−4^ by using higher diffraction orders and correcting the scattering intensity variations across the analyzer surface. This is not sufficient for efficient high-energy-resolution XES experiments which require an energy resolution comparable with or better than the natural linewidths of the emission lines and high count rates. For example, the Nb *K*α line at around 16.5 keV has a natural linewidth of ∼6 eV and a spectrometer with Δ*E*/*E* ≃ 10^−3^ would correspond to 16 eV resolution, almost three times larger than the natural linewidth (Campbell & Papp, 2001 ▸). Recently, high-photon-energy XES measurements using Laue analyzers with high energy resolution were reported by Ravel *et al.* (2018 ▸) and Jagodziński *et al.* (2019 ▸). In these works the Laue crystal was bent to a cylindrical shape and the DuMond geometry was used (DuMond, 1947 ▸).

**Figure 1:**
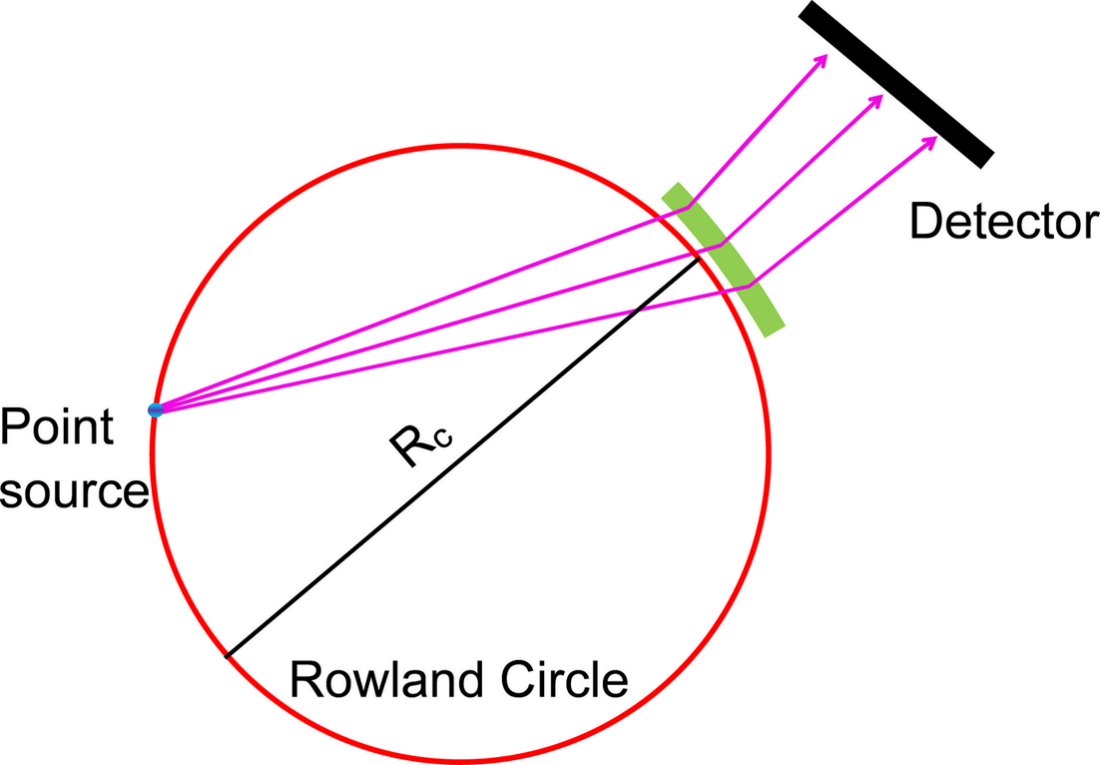
Schematic of the DuMond geometry. The Laue analyzer is bent to a cylindrical shape with curvature of *R*_C_. The light is diffracted by crystal planes oriented in the same direction as the incoming beam, instead of perpendicular planes as in Bragg geometry. The emission is collected from a point-like source and imaged on a detector. When operating in on-Rowland geometry the source and the Laue crystal are located exactly on the Rowland circle and the distance is *R*_C_. When operating in off-Rowland geometry, the Laue analyzer has an offset from the Rowland circle. The detector is behind the analyzer.

There are two working geometries for Laue analyzers, the Cauchois type (Cauchois, 1932 ▸) for extended sources and the DuMond type for point-like sources. The DuMond geometry can be regarded as an inverse Johann geometry, as shown in Fig. 1 ▸, where the diffracted light passing through the crystal does not converge but diverges onto the detector. Therefore, specific characteristics of the Laue analyzer should be considered as they can induce distortions on the spectral image on the detector. Compared with reflection geometry, the transmission geometry requires an open aperture on the crystal support, which can induce a saddle-like distortion in the analyzer surface due to anisotropic Poisson-ratio effects (Lethbridge *et al.*, 2010 ▸) and worse surface distortion on the edge of the open area. Furthermore, the emission image on the detector is sensitive to distortions in the analyzer surface due to the unfocused characteristic of the DuMond geometry (see Fig. 1 ▸). Those distortions can result in complex emission images with a consequent effect in final energy resolution. Both Laue spectrometers described by Ravel *et al.* (2018 ▸) and Jagodziński *et al.* (2019 ▸) operate in the off-Rowland condition of the DuMond geometry to mitigate the effects arising from the analyzer surface distortion. However, different working modes are employed in the measurements: Jagodzinksi *et al.* used linked scans and Ravel *et al.* employed dispersion mode.

The development of the high-energy Laue emission spectrometer at FXE started with an investigation of the optimum analyzer thickness and the effects of asymmetric angles on the diffraction efficiency. Different geometries were evaluated through X-ray tracing simulations, which were carried out using *SHADOWOUI* of the *OASYS* suite (Rebuffi & Rio, 2017 ▸; Rebuffi & Sanchez del Rio, 2016 ▸). This allowed additional optimization of key design parameters to improve the spectrometer efficiency and simplify the image processing, while providing routes for a final energy resolution compatible with the emission measurement requirements. Guided by our X-ray tracing simulations and considering the pump–probe character of the experiments at FXE and specifics of XFEL operation, the energy-dispersive mode proved to be more appropriate and was chosen for the Laue spectrometer presented here.

### 2.1. X-ray transmission optimization

**Figure 2:**
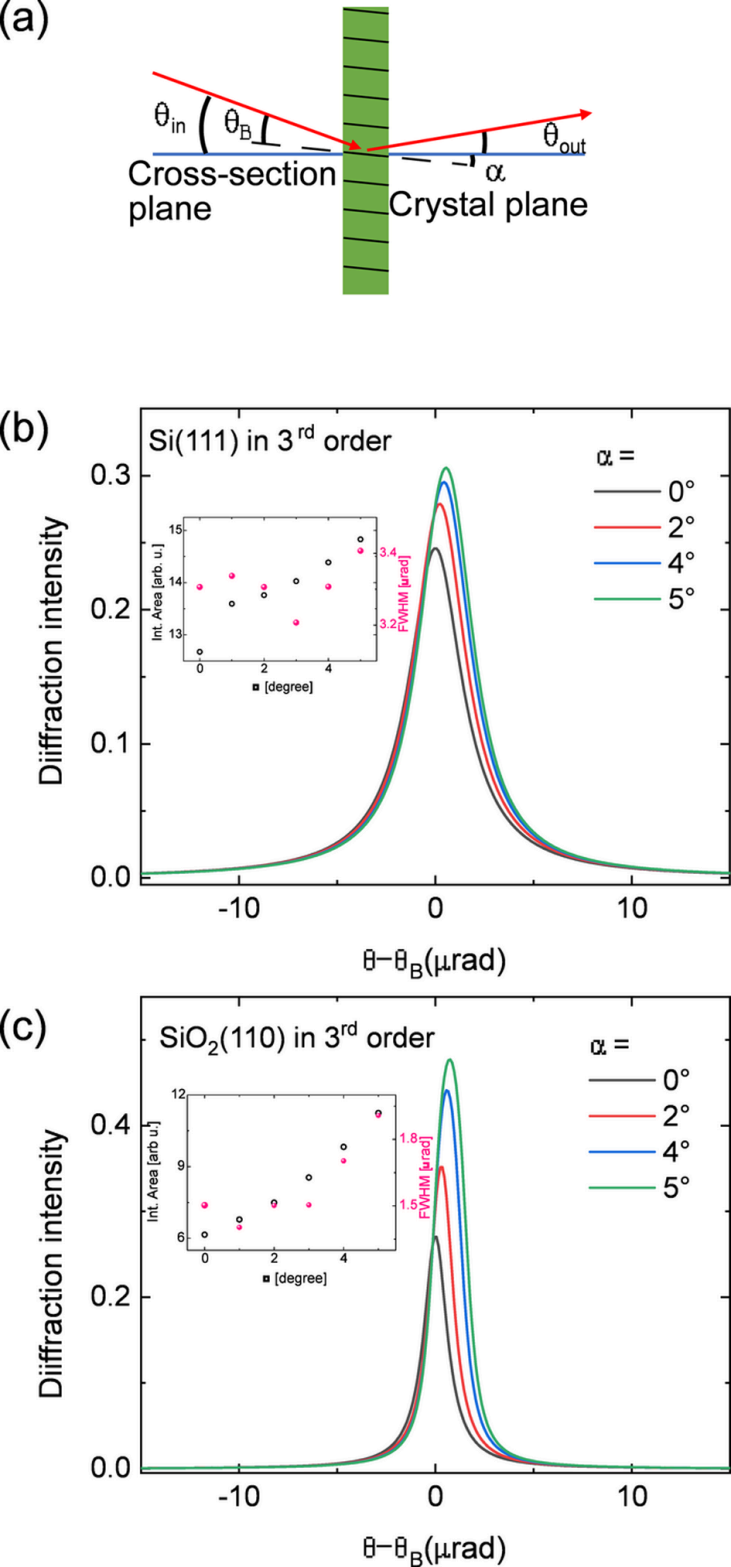
(*a*) Schematic of asymmetric cutting for the Laue crystal. The asymmetric angle α is defined as the cross angle between the crystal and cross-section planes. When an asymmetric angle is present, the incidence angle θ_in_ = θ_B_ + α, where θ_B_ is the Bragg angle. θ_in_ + θ_out_ is still 2θ_B_, so the asymmetric cutting will not affect the geometry for the detector. Calculated rocking curves at different asymmetric cutting angles for (*b*) Si(333) and (*c*) SiO_2_(330). The integrated area and FWHM are shown in the insets. An example energy of 18.625 keV is considered in the calculations.

The Laue spectrometer is expected to operate in the >15 keV range and, given that the standard minimum thickness of commercial perfect crystals is usually limited to several hundreds of micrometres, the X-ray transmission of Laue analyzers can be a limiting factor. For example, Jagodziński *et al.* (2019 ▸) employed a silicon crystal with 0.5 mm thickness, which translates into ∼20% diffraction efficiency at ∼19 keV. Here, we have chosen silicon (111) and quartz (110) crystals as the Laue analyzers for the spectrometer, with thicknesses of 300 µm and 400 µm, respectively. One way to increase the diffraction efficiency given a fixed crystal thickness is to use asymmetric cuts. Fig. 2 ▸(*a*) illustrates the asymmetric cut in Laue analyzers, referring to the presence of an angle α between the physical cross-section plane and the crystal planes. In general, this can tune the effective diffraction efficiency of the crystal and also the angular acceptance. In this work, the definition of α for Laue crystals differs from that for Bragg crystals, with a 90° rotation offset between them (see Fig. 2 ▸). In general, both the diffraction intensity and the Darwin width increase with increasing α for Laue crystals, meaning that a careful choice of the asymmetric angle is required to prevent degradation of the energy resolution.

The evaluation of the influence of varying crystal thickness and asymmetric cutting angles was performed using dynamic diffraction calculations carried out using the *XOP* package (Sanchez del Rio & Dejus, 2011 ▸). Figs. 2(*b*) and 2(*c*) show the calculated rocking curves for the third-order diffraction of silicon (111) and quartz (110) crystals, respectively. These could be used for measuring niobium *K*β XES (∼18.62 keV). As expected, compared with the symmetrically cut case (α = 0°) the diffraction intensity increases with increasing α. For the Si(333) diffraction, the X-ray tracing simulations show that as α increases from 0° to 2°, the diffraction intensity increases from around 0.25 to 0.28 with a negligible increase in the full width at half-maximum (FWHM) of the rocking curve, going from 3.3 µrad to 3.4 µrad with α = 0° and 2°, respectively. As α increases further, this intensity enhancement becomes less significant and the main effect is a further broadening in the Darwin width, as shown by the similar Si(333) rocking curves in Fig. 2 ▸(*b*) with α = 4° or 5°. A similar behavior is also observed for the SiO_2_(330) diffraction, as shown in Fig. 2 ▸(*c*). Compared with Si, SiO_2_ exhibits a more pronounced sensitivity to the asymmetric cut. This is likely due to the SiO_2_ crystal having more scattering centers within its unit cell contributing to the diffraction, resulting in stronger interference effects. These results indicate that using relatively small asymmetric angles α results in a noticeable increase in the diffraction efficiency. The associated increase in the Darwin widths can further improve the diffraction efficiency. On the other hand, if the Darwin width becomes too large the energy resolution can be affected, so a compromise needs to be found. This increase in Darwin width due to asymmetric angles is more significant for higher-order diffraction indices, *e.g.* fifth order for Si(111) and fourth order for SiO_2_(110) (Shvyd’Ko, 2004 ▸). We have chosen α = 2.5° and 3.0° for the silicon and quartz analyzers, respectively. The Darwin widths for both analyzers are <5 µrad, which, based on an estimation for the energy resolution of Δ*E* = 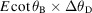, with Δθ_D_ being the Darwin width, leads to a contribution of less than 0.3 eV to the energy resolution at the Nb *K*β energy (18.62 keV).

### 2.2. Laue geometry and X-ray tracing simulations

The Laue analyzer can be bent into either a logarithmic spiral or a simple cylindrical shape. These are used to address the mismatch between the narrow Darwin width of a perfect crystal and the intrinsic nature of the 4π divergence of emission. The analyzer with a logarithmic spiral shape operating in Rowland geometry can, in principle, collect a single photon energy emitted from a point source across the entire area of the analyzer and minimize the background scattering. However, the energy resolution is affected by the commonly observed distorted diffraction images. X-rays with different energies originating from the same area on the crystal may overlap in the detector, leading to insufficient energy resolution for emission spectrum measurements. Thus, analyzers with a logarithmic spiral are typically employed for XAS detection in fluorescence geometry (Zhong *et al.*, 1999 ▸; Kropf *et al.*, 2003 ▸; Kropf *et al.*, 2005 ▸; Wakisaka *et al.*, 2017 ▸), rather than emission spectrum measurement. The complexity of those diffraction images may be attributed to pronounced distortions caused by variations in the local bending radius from one analyzer end to the other. The simpler cylindrical shape likely results in less distorted surfaces. However, since the Laue analyzer operates in defocused geometry, the diffraction image is more sensitive to surface distortions and aberrations, in comparison with the Bragg crystals. The challenges posed by complex diffraction images cannot be solved by simply modifying the bending geometry. Instead, a suitable working mode is required. For example, the off-Rowland condition was employed on other spectrometers using Laue analyzers (Ravel *et al.*, 2018 ▸; Jagodziński *et al.*, 2019 ▸) in order to have a more uniform image. On the other hand, the magnitude of the bending curvature can also significantly affect the image. For instance, a complex ‘S’-shaped stripe was observed with a bending curvature of 0.5 m by Ravel *et al.* (2018 ▸), while a simpler single line stripe was observed in the detector with a bending curvature of 2.0 m by Jagodziński *et al.* (2019 ▸).

**Figure 3:**
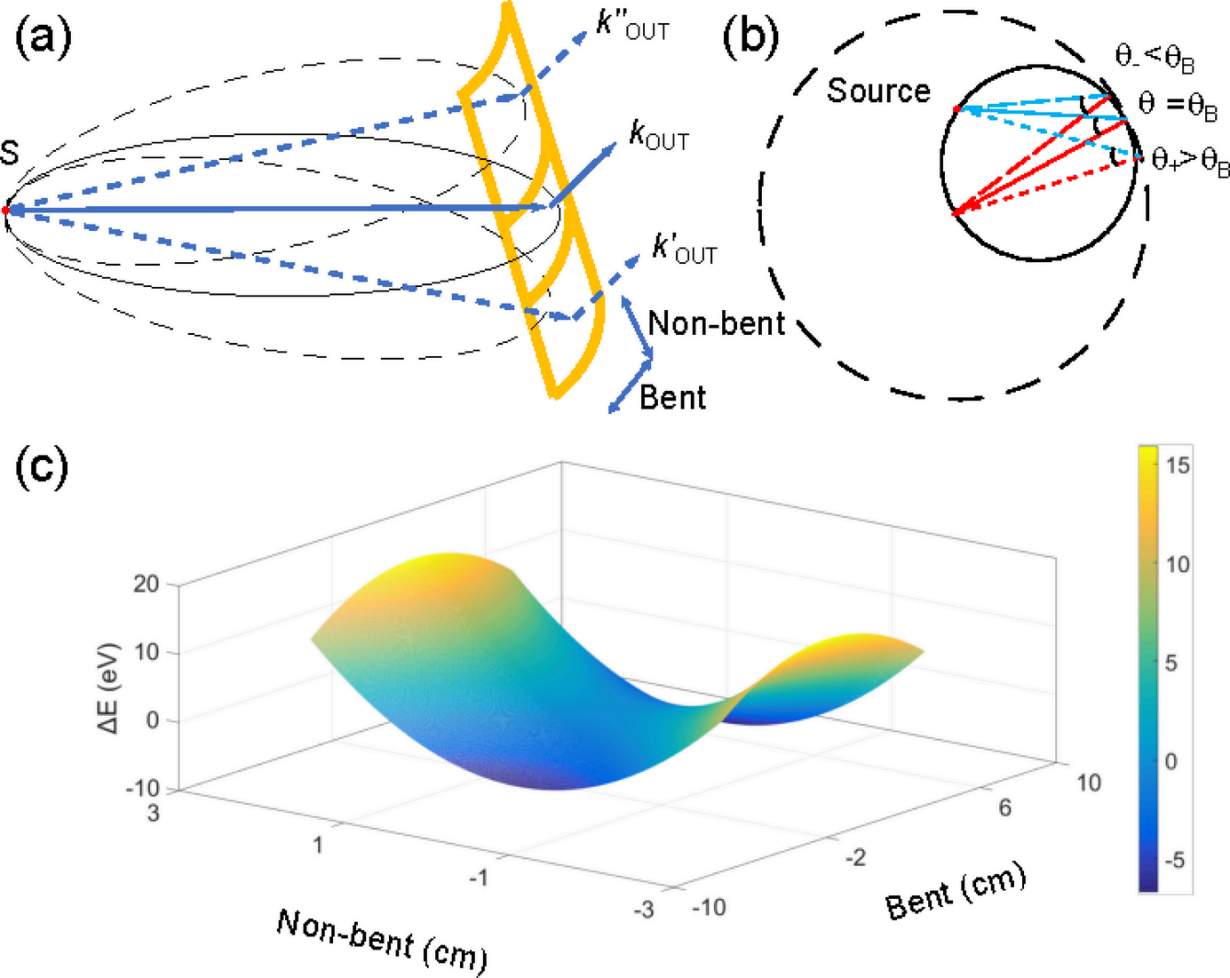
Calculated dispersion of the cylindrical shape analyzer working on the Rowland circle condition. Panels (*a*) and (*b*) illustrate the schematics of the aberrations in the bent and non-bent directions. In (*c*) the 2D dispersion map after converting the angular difference to the energy difference is shown.

Laue analyzers with a cylindrical shape are able to produce dispersion behavior even when working in exact Rowland geometry. This dispersive ability arises from analyzer aberrations both in the bent and the non-bent directions, as illustrated in Fig. 3 ▸. In the non-bent direction there are different angles and distances between the (point) source and analyzer surface on both the upper and lower analyzer parts with respect to the center region. For the bent direction, the emitted X-rays also hit the crystal surface at different angles because of the mismatch between the bent curvature and the Rowland circle. A similar concept was also realized using Bragg analyzers in combination with position-sensitive pixel detectors to improve the energy resolution (Huotari *et al.*, 2005 ▸; Szlachetko *et al.*, 2012 ▸; Alonso-Mori *et al.*, 2012 ▸; Moretti Sala *et al.*, 2018 ▸; Huotari *et al.*, 2017 ▸). Given the well determined geometry of the relative positions of the crystal surface and the point source, the angular difference across the whole analyzer area can be calculated, providing a map of the different analyzer regions collecting different energies. To illustrate this we have performed X-ray tracing simulations of 18625 eV (Nb *K*β_1_) photons diffracting from a Si(333) Laue analyzer with 155 cm curvature in on-Rowland geometry, converting the angular difference to energy difference, as shown in the 2D mesh in Fig. 3 ▸(*c*). The central region analyzes energies with the smallest difference with respect to the central energy. On the other hand, the edge regions in both bent and non-bent directions exhibit larger dispersion, with a maximum energy difference of about 20 eV if considering an analyzer with dimensions of 5 cm × 10 cm. This would be insufficient to measure a full *K*β_1,3_ emission spectrum of a 4*d* metal, which spans around 100 eV. Moreover, the areas corresponding to different analyzed energies are not uniform, resulting in varying intensities for different energies. This inconsistency makes emission spectra measured using Laue analyzers in on-Rowland geometry challenging to analyze and unsuitable for applications envisioned at the FXE instrument.

**Figure 4:**
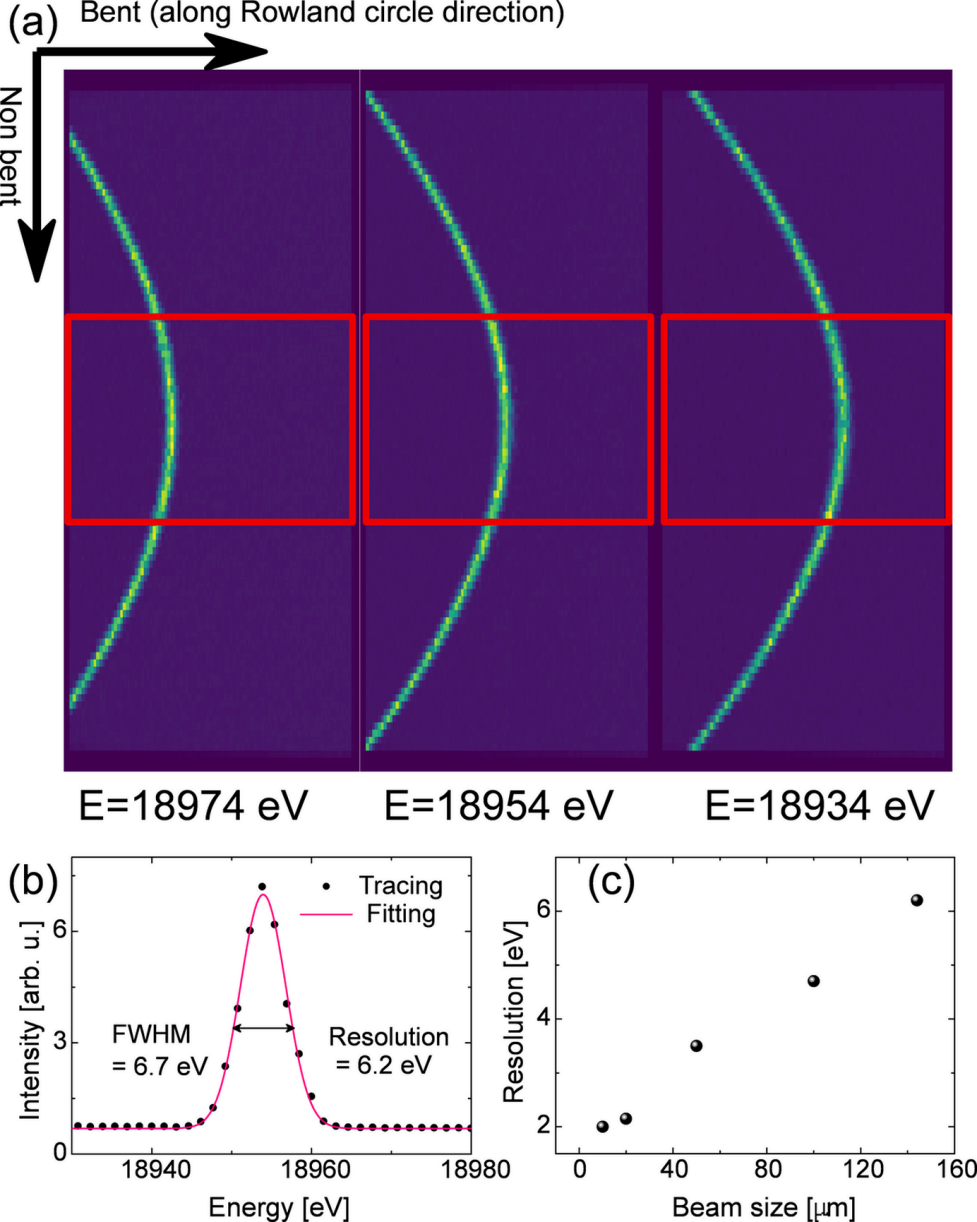
(*a*) Calculated emission images of a Laue analyzer for different photon energies when working under the off-Rowland condition. A point-like source with beam size of 100 µm × 100 µm in FWHM, energy resolution of 1.4 × 10^−4^ (Δ*E*/*E*, where Δ*E* is defined by FWHM) and varying incoming beam central energies were considered as the emitted source. A Si(333) Laue analyzer with a cylindrical shape and a bent curvature of 155 cm was used. Surface distortions were omitted here to simplify the X-ray tracing. The analyzer is positioned 5 cm farther from the Rowland circle. The red boxes in the figures represent a realistic size of a Laue analyzer. (*b*) Energy resolution evaluation using a beam size of 144 µm. A 15 mm-wide region of interest (ROI) at the central position is selected to project the elastic scattering spectrum. A series of Gaussian fittings are performed to extract the peak positions, followed by polynomial fitting to determine the position-to-energy calibration. The energy resolution of the analyzer is then obtained by deconvoluting the Gaussian functions. (*c*) The energy resolution as a function of beam size is determined using the same procedure described in (*b*) for various beam sizes.

A simpler dispersive behavior can be obtained for Laue analyzers when they are located in an off-Rowland circle geometry. This approach has been previously used to collect emission spectra for niobium compounds (Ravel *et al.*, 2018 ▸). Fig. 4 ▸ shows the calculated emission images corresponding to different energies when a Laue analyzer with 155 cm radius is placed 5 cm off from the Rowland circle. Each emission image corresponding to a single energy is a unique bent stripe that moves along the bent direction and slightly changes its curvature with changing photon energy. The non-bent plane also exhibits energy dispersion, as indicated by the curved emission image. However, for a realistic Laue analyzer with limited size, *e.g.* 8 cm × 3 cm (bent and non-bent directions, respectively) the emission stripe becomes simpler, as shown by the signal inside red boxes in Fig. 4 ▸. Within the analyzer area, the shape of the emission stripe approaches a single line and moves along the bent direction. Additionally, in this configuration the energy bandpass at about 18.9 keV is close to 80 eV, being large enough to allow dispersive measurements of the complete emission spectrum. Based on the tracing results of on- and off-Rowland conditions, one can imagine that if the analyzer is positioned on the Rowland circle, the image becomes diffuse and is more significantly affected by surface distortions. Conversely, if the analyzer is positioned off the Rowland circle, the image becomes more localized and is less affected by surface distortion. Therefore, the off-Rowland geometry provides a reasonable solution to mitigate the effects arising from surface distortion which is typically a problem of Laue analyzers.

When X-ray optics work in dispersive mode, a compromise needs to be found between their efficiency and energy resolution. In principle, the shorter bent curvature leads to a larger solid angle of collection, resulting in better efficiency. However, it also results in worse energy resolution because the emitted source cannot be a point without dimensions. In pump–probe experiments at the FXE instrument of the European XFEL, the desired beamsize varies from tens of micrometres to hundreds of micrometres depending on different sample and experimental conditions. In this range, the ratio of source size to distance is typically the primary contributor to the energy resolution. Therefore, the bent curvature needs to be carefully evaluated to ensure compatibility with the XES measurements of 4*d* metals with the *K* edge in the 15–26 keV range, where the natural linewidths of emission lines vary from about 5 eV to 10 eV. The example tracing images are shown in Fig. 4 ▸(*a*); a beam size of l00 µm was considered in the tracing simulation presented here. The photon energy was set to 18953.4 eV, corresponding to the Nb *K*β_2_ emission and an energy resolution of 1.4 × 10^−4^ was chosen to create a Gaussian-distributed monochromatic beam from a Si(111) double crystal. A Si(333) Laue analyzer with a cylindrical curvature and a bent radius of 155 cm was chosen for X-ray tracing. Distortions on the analyzer surface were not considered in order to simplify the X-ray tracing. The analyzer is positioned 5 cm away from the Rowland circle to produce sufficient energy dispersion to cover the Nb *K*β_2_ emission. The theoretically expected energy resolution is depicted in Fig. 4 ▸(*b*). The central region is selected to project the elastic scattering spectrum. A series of Gaussian fittings are performed to extract the peak positions, followed by polynomial fitting to determine the position-to-energy calibration. The energy resolution of the analyzer is then obtained by deconvoluting the Gaussian functions. For example, the tracing results in 6.2 eV FWHM when using a 144 µm point-like source (to account for the 45° projection in the sample of an initial 100 µm beam). In the experimental tests (Section 4), a slightly worse resolution (∼6.7 eV) was obtained when using a similar beam size. We attribute this to contributions from analyzer surface distortions, crystal strain and effects of the detector pixel size, all of which are omitted in the simulations. When the beam size is decreased to 100 µm, the expected energy resolution goes down to 4.7 eV FWHM, which is sufficient for measuring high-resolution niobium XES. As shown in Fig. 4 ▸(*c*), the resolution can ideally be improved to better than 2 eV by reducing the beam size to below 10 µm. The energy resolution can be further improved by using higher Bragg angles, *i.e.* higher diffraction indices. However, the narrower Darwin widths associated with higher diffraction indices will also result in lower efficiency. In the X-ray tracing simulations we have chosen relatively moderate diffraction indices, *e.g.* Si(333) and SiO_2_(330). This allowed us to first optimize the bent curvature and carry out an initial evaluation of the practical aspects of operation.

### 2.3. Analyzer design

**Figure 5:**
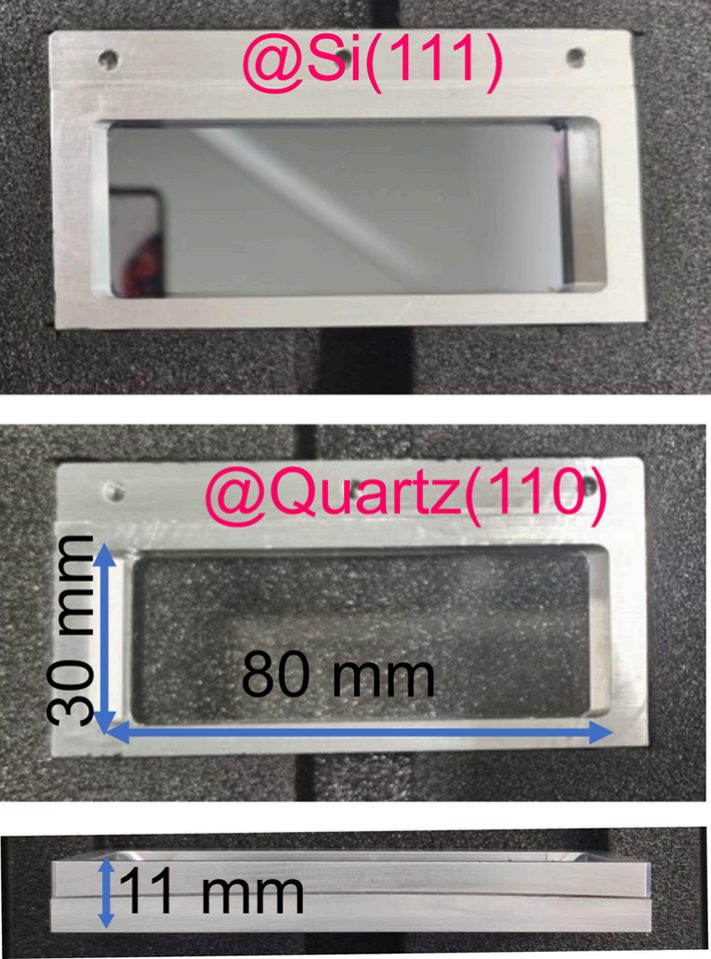
Top and side views of the photographs of Laue analyzers. The top panel depicts silicon (111) and the middle panel depicts quartz (110). The aperture size is 80 mm in the bent direction and 30 mm in the non-bent direction. The bottom panel shows a side view of the bender frame. The crystal curvature of ∼150 cm is visible.

The X-ray tracing simulations guided the choice of the most appropriate analyzers for the Laue spectrometer. The analyzer design profits from advances in manufacturing technologies that allowed the use of curvatures of relatively short radius and a fixed radius by using a newly engineered support frame. A compromise was found by using a ∼150 cm bending curve, which proved to be sufficiently large to not induce noticeable surface distortion as observed when using a 50 cm radius analyzer (Ravel *et al.*, 2018 ▸). Reducing the radius from 2 m used in the dynamically bent analyzers (Jagodziński *et al.*, 2019 ▸) to the chosen ∼150 cm results in an increase in collection efficiency by a factor of about 1.8. The analyzer support frame was engineered to have thin walls (∼11 mm), providing a large clear aperture of 80 cm × 30 cm in the bent and non-bent directions, respectively. Note that the crystal will be subjected to saddle-like distortions, even though the frame is cylindrical only in one direction. In the spectrometer reported by Jagodzinksi *et al.*, a large area of analyzer was shadowed by the crystal bender; thus our design results in a usable area comparable with that of the much larger dynamically bent analyzer (Jagodziński *et al.*, 2019 ▸). The thinner support walls also enable working at larger diffraction angles (∼50°) needed for higher diffraction indices [*e.g.* (777) order in the case of Si and (440) order for quartz]. A common issue observed on dynamic benders is the variation in the bending radius due to non-uniform forces on the crystal surface, particularly on the edges. Thanks to its optimized design combining a compact crystal in a sturdy frame and large opening angles, this effect is not observed in our static bender. Additionally, operating analyzers with fixed radius simplifies the spectrometer alignment and operation, while also reducing costs and risks (the analyzer can break if bent excessively) associated with the dynamic bender mechanism. Fig. 5 ▸ shows photographs of our silicon and quartz Laue analyzers using the same design for the bender frame with different curvatures. The Si analyzer has a radius of 150 cm and the quartz one 155 cm.

## 3. The Laue spectrometer

**Figure 6:**
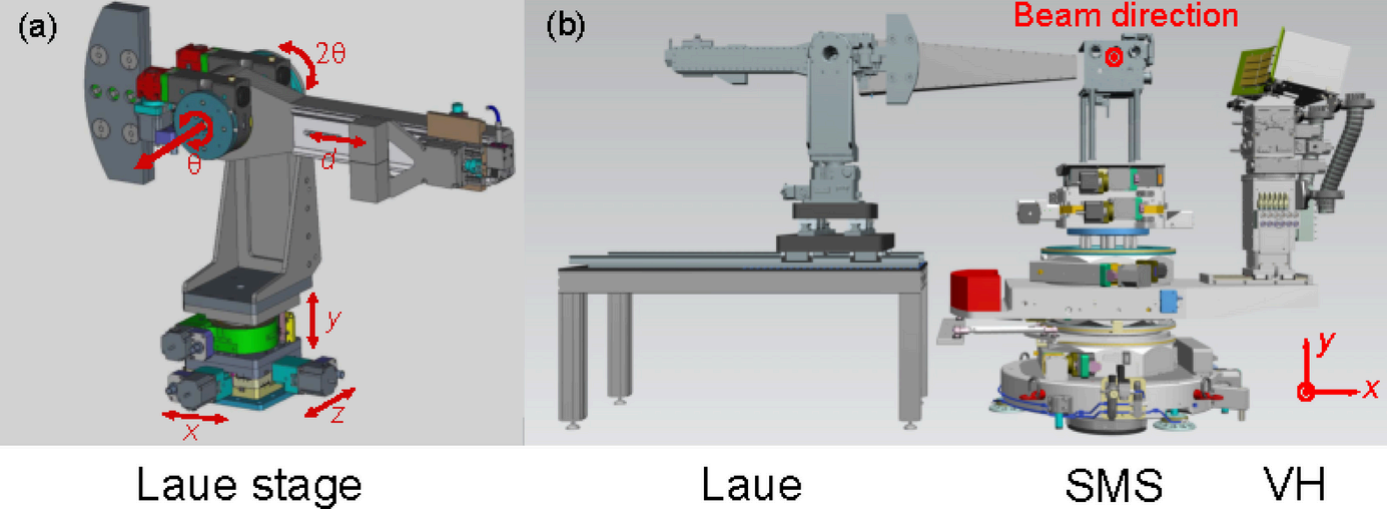
(*a*) 3D drawing of the motorized stages for the Laue spectrometer installed at the FXE instrument. The Laue analyzer is mounted at the center of the θ stage and the detector is placed in the 2θ arm. (*b*) The layout of the Laue spectrometer, SMS and von Hamos (VH) spectrometer used in measurements at FXE is shown in the view along the beam direction.

The Laue spectrometer was designed to operate in the horizontal scattering plane, which can be varied between 0° and 90°. At very high X-ray energies the elastic and Compton scatterings usually contribute significantly to the background in the emission signal; thus working at 90° scattering angle is preferred for most experiments. During operation at the FXE instrument the Laue spectrometer is docked into the sample-mounting stack (SMS), perpendicular to the beam propagation direction in place of the Johann spectrometer (see Fig. 6 ▸). However, the backward scattering geometry was chosen during the test at SuperXAS due to lateral space constraints in the experimental hutch. The JUNGFRAU detector was used for the spectrometer commissioning and mounted on the 2θ arm. In this geometry, the free space in the forward scattering direction leaves the possibility for other equipment to perform parallel measurements, *e.g.* inelastic X-ray scattering (Ament *et al.*, 2011 ▸), high-resolution Compton scattering (Suortti *et al.*, 1999 ▸; Itou *et al.*, 2001 ▸; Hiraoka *et al.*, 2001 ▸; Hiraoka *et al.*, 2005 ▸), or X-ray diffraction and scattering using the LPD-1M detector (Galler *et al.*, 2019 ▸; Khakhulin *et al.*, 2020 ▸). The latter is particularly advantageous for combining high-energy scattering and spectroscopy measurements simultaneously. The Laue spectrometer can also operate simultaneously with the von Hamos spectrometer, enabling multi-elemental spectroscopic studies on materials containing *e.g.* 3*d* and 4*d* elements, as well as providing a direct efficiency comparison between measurements taken with Laue and Bragg analyzers.

### 3.1. Spectrometer stage

The dispersive direction of our Laue analyzer was chosen to be on the vertical plane to minimize degradation of energy resolution caused by practical operation considerations, *e.g.* the beam footprint in grazing-incidence experiments and the horizontal beam jitter lead to a large effective beam size, increasing their contribution to the energy resolution. A custom-made set of stages from Huber is used as the spectrometer platform. A drawing of the Laue spectrometer and all its stages and degrees of freedom is shown in Fig. 6 ▸(*a*). The θ and 2θ stages are used to rotate the analyzer and detector arm, respectively. The θ motor can operate over a full 360° angular range, and the rotation motors are elevated on top of linear motors to allow a wide working range of −60°/+240°. Three orthogonal linear motors along *X*, *Y* and *Z* directions are used to precisely align the analyzer. A long rail on the 2θ arm with a total length of 440 mm is equipped with a motorized stage with a travel range of ±30 mm. It is used to control the distance from the analyzer to the detector. The complete stage is placed over a long rail sitting on a 1.6 m-long table, allowing large variations in the sample–analyzer distance. Absolute encoders for precise alignment and scan are used on the *X*, *Z*, θ and 2θ movements.

## 4. Commissioning at the synchrotron

The first Si(111) Laue analyzer prototype was tested on a commissioning campaign conducted at the SuperXAS beamline at the Swiss Light Source (SLS) in Switzerland, where a Laue spectrometer with a dynamically bent analyzer is installed (Jagodziński *et al.*, 2019 ▸). The measurements aimed at determining the improvements in overall efficiency and the energy resolution, while also investigating the possibility of using the Laue analyzer out of the Rowland circle to explore the dispersive capabilities. For this we have chosen to measure non-resonant Nb *K*β XES and benchmark our results against those reported previously (Ravel *et al.*, 2018 ▸). Space constraints in the SuperXAS hutch limited the tests of our Laue analyzer to either forward or backward scattering geometries. Considering the influence of strong Compton scattering at high energies on the X-ray emission signals, the backward scattering geometry is the best choice under the given conditions at SuperXAS, where the Compton peak shifts to lower energies at large scattering angles. In these measurements a scattering angle of ∼167° was used. In this setup the θ–2θ rotations are on the horizontal plane, meaning that the dispersion axis of the analyzer must also be on the horizontal plane. A Pilatus 100K-S detector was used to detect the emission signals. The 1 mm-thick silicon sensor improves the detector efficiency at high energies. Further details on the spectrometer stage and detector can be found in Jagodziński *et al.* (2019 ▸).

Niobium *K*β and valence-to-core (VtC) emission at about 18.62 keV and 18.96 keV, respectively, were chosen for the test measurements. These emission lines can be analyzed using the (333) diffraction of a Laue silicon crystal placed at a Bragg angle of about 18.6° (*K*β_1_) and 18.2° (VtC). Once the Laue spectrometer was set to these conditions and the analyzer placed on the Rowland circle, the incoming beam was tuned to 18.6 keV and the elastic scattering from an aluminium target was used to align the spectrometer. The analyzer was then moved approximately +16 mm away from the Rowland circle position in order to verify the energy dispersion capabilities. This was done by using a 250 µm-thick Nb foil as sample and setting the incoming energy to 19.1 keV, *i.e.* above the Nb *K* edge.

**Figure 7:**
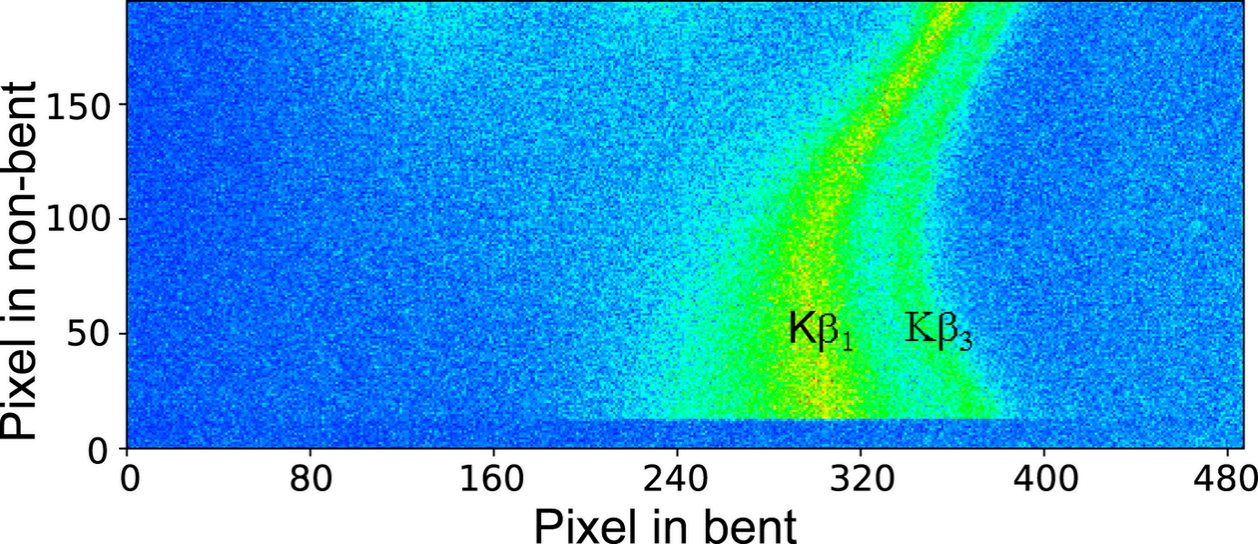
Representative Pilatus 100K-S detector image of the Nb *K*β_1_ and *K*β_3_ XES. In this measurement the incoming X-ray beam was set to 19.1 keV to excite the emission of a 250 µm-thick niobium foil. An integration time of 180 s was used.

Fig. 7 ▸ shows an example emission image from these measurements. Two well separated structures corresponding to the Nb *K*β_1_ and *K*β_3_ lines can be clearly identified, suggesting that in this configuration the energy resolution is sufficient to resolve the two peaks which are separated by about 15.5 eV (Elam *et al.*, 2002 ▸). The energy dispersion is mainly along the bent axis, with the energy increasing from right to left in Fig. 7 ▸. Note that the separation between the *K*β_1_ and *K*β_3_ emission lines in Fig. 7 ▸ is different in the top and bottom regions on the image, indicating that the energy dispersion varies across different non-bent regions of the analyzer. Both stripes are curved, consistent with the prediction from the X-ray tracing simulation. The data in Fig. 7 ▸ demonstrate two basic characteristics of the Laue analyzers operating on the off-Rowland circle condition: the energy dispersion along the bent direction and the defocused geometry in the non-bent axis due to crystal surface distortions. These result in rather complex images and special image processing procedures are needed to convert the XES images into spectra. The simple projection along the dispersive axis and linear energy calibration procedures commonly used in data from von Hamos spectrometers (Hoszowska *et al.*, 1996 ▸) are not adequate to process the detector images of a Laue analyzer in DuMond geometry.

### 4.1. Energy calibration

Elastic scattering from a monochromatic beam is often used to calibrate the energy axis of spectrometers operating in dispersive mode. This same concept was employed in the measurements using our Laue analyzer at the SuperXAS beamline. Briefly, elastic scattering data at different monochromatic energies are collected using the sample itself as the scattering source. This has the advantage of keeping the spectrometer source fixed and acquiring elastic scattering for each sample measured. The incoming energy was set to the vicinity of the Nb *K*β emission and varied in steps of 2 eV. Each data point required about 30 s of integration time to provide sufficient signal levels for performing the energy calibration. Once a complete data set of elastic scattering was collected, different image processing methods were used to generate the XES spectra. These circumvented the non-linearity issues in the calibration curves and provided a correction for the distorted emission image shapes.

#### 4.1.1. Algorithm 1: calibration mask

**Figure 8:**
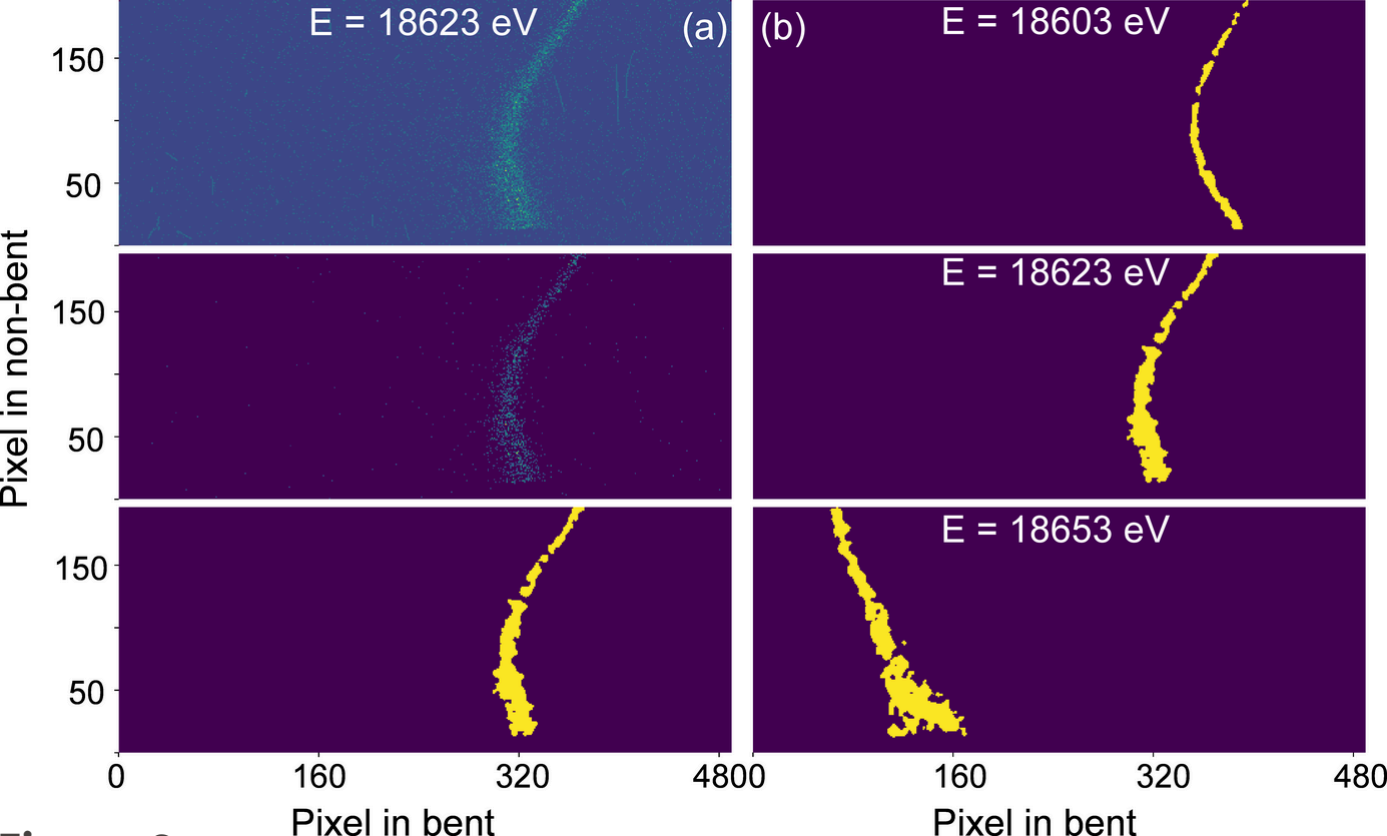
(*a*) Mask preparation from the raw image of elastic scattering (top panel). A threshold is applied to obtain the middle panel, then median and Gaussian filtering are applied to get the Boolean matrix (bottom panel), *i.e.* the mask image. (*b*) Example masks for different energies collected from elastic scattering.

One method to perform the spectrometer energy calibration is referred to as the ‘mask’ algorithm. This method, first proposed by Ravel *et al.* (2018 ▸), uses the elastic scattering data set to create a series of image masks for each energy. The masks can then be applied to the XES data and used to extract emission intensities at each unique energy, which are finally merged to form the emission spectrum. Fig. 8 ▸(*a*) illustrates the basic steps needed to prepare the image mask starting from the elastic scattering data. First, an appropriate intensity threshold is set to distinguish between signal and background in the raw image (top panel), resulting in a cleaner image (middle panel); however, some noisy pixels remain due to the long integration times needed to acquire the elastic data. In a next step, a median filtering is applied to further reduce the residual noise and improve the signal-to-background ratio. This is followed by a Gaussian filtering to further remove the noise and improve the continuity of the signal area. After the threshold subtraction and filtering are applied, each pixel on the detector image corresponding to a given energy is assigned a binary value depending on whether it contains signal (set to 1, or True) or not (set to 0, or False). This Boolean logic 2D array is the so-called ‘mask’ and the final result for a single energy is shown in the bottom panel of Fig. 8 ▸(*a*). This procedure is applied to all elastic scattering images making up the energy calibration data set, creating a set of masks, one for each energy. Fig. 8 ▸(*b*) shows the example masks at different energies. The distorted stripe is clearly shown for example in the bottom panel. The behavior of the elastic scattering is consistent with the X-ray tracing simulations and that observed in the emission images: the elastic scattering stripes shift from right to left in the bent direction with increasing incident energy, while their shape and curvature also vary slightly. The complete set of masks is finally used to construct the XES spectrum by multiplying it by the emission image and summing the intensity of all pixels at a given energy.

**Figure 9:**
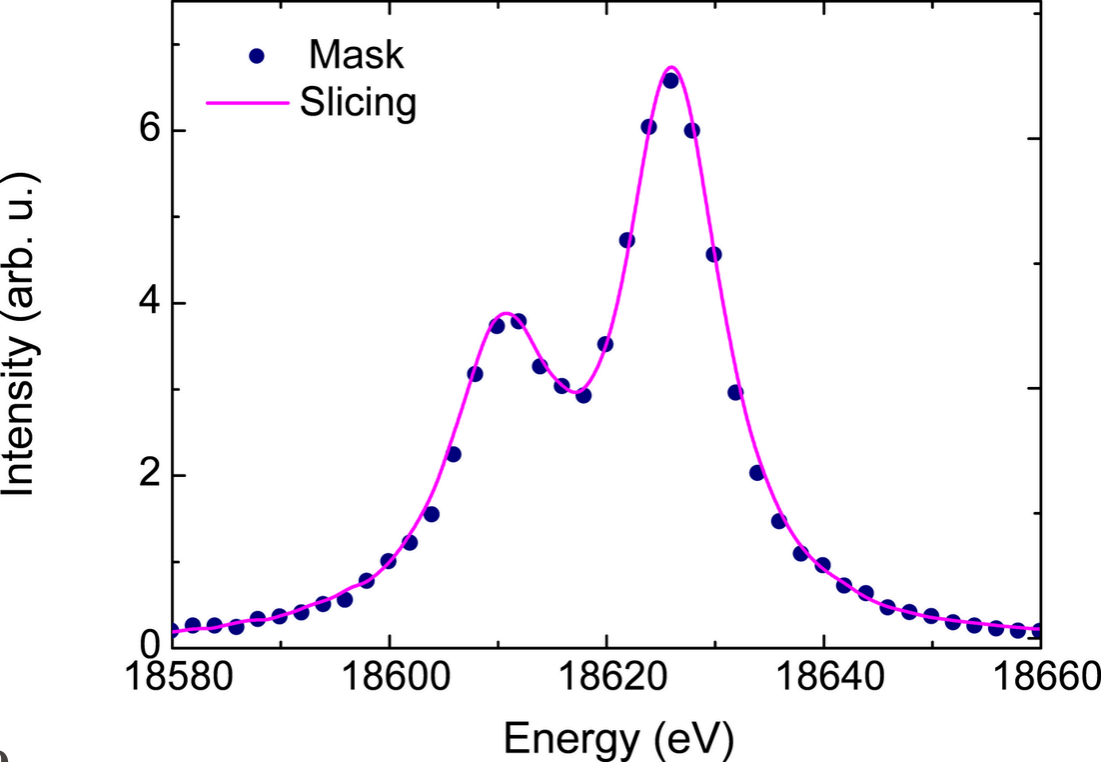
The final *K*β_1_ and *K*β_3_ XES spectrum of a 250 µm niobium foil. The background is removed for better comparison. The comparison between the mask and slicing/re-binning algorithms shows similar results.

Fig. 9 ▸ depicts the *K*β_1_ and *K*β_3_ XES spectrum of a 250 µm-thick niobium foil converted from the emission image using the mask algorithm (blue dots). This attests to the effectiveness of the mask algorithm in reconstructing XES spectra from emission images, even for distorted image shapes. Importantly, the energy step in the final spectrum is limited by the sparsity used in the calibration data set. In the elastic scattering measurements presented here, an energy stepping of 2 eV was used, which directly reflects the relatively coarsely sampled spectrum shown in Fig. 9 ▸. Note that due to the better design and surface quality of our Laue analyzers, the emission images are less complex than the ‘S’ shape obtained by Ravel *et al.* (2018 ▸), enabling more straightforward image processing methods.

#### 4.1.2. Algorithm 2: slicing and re-binning

**Figure 10:**
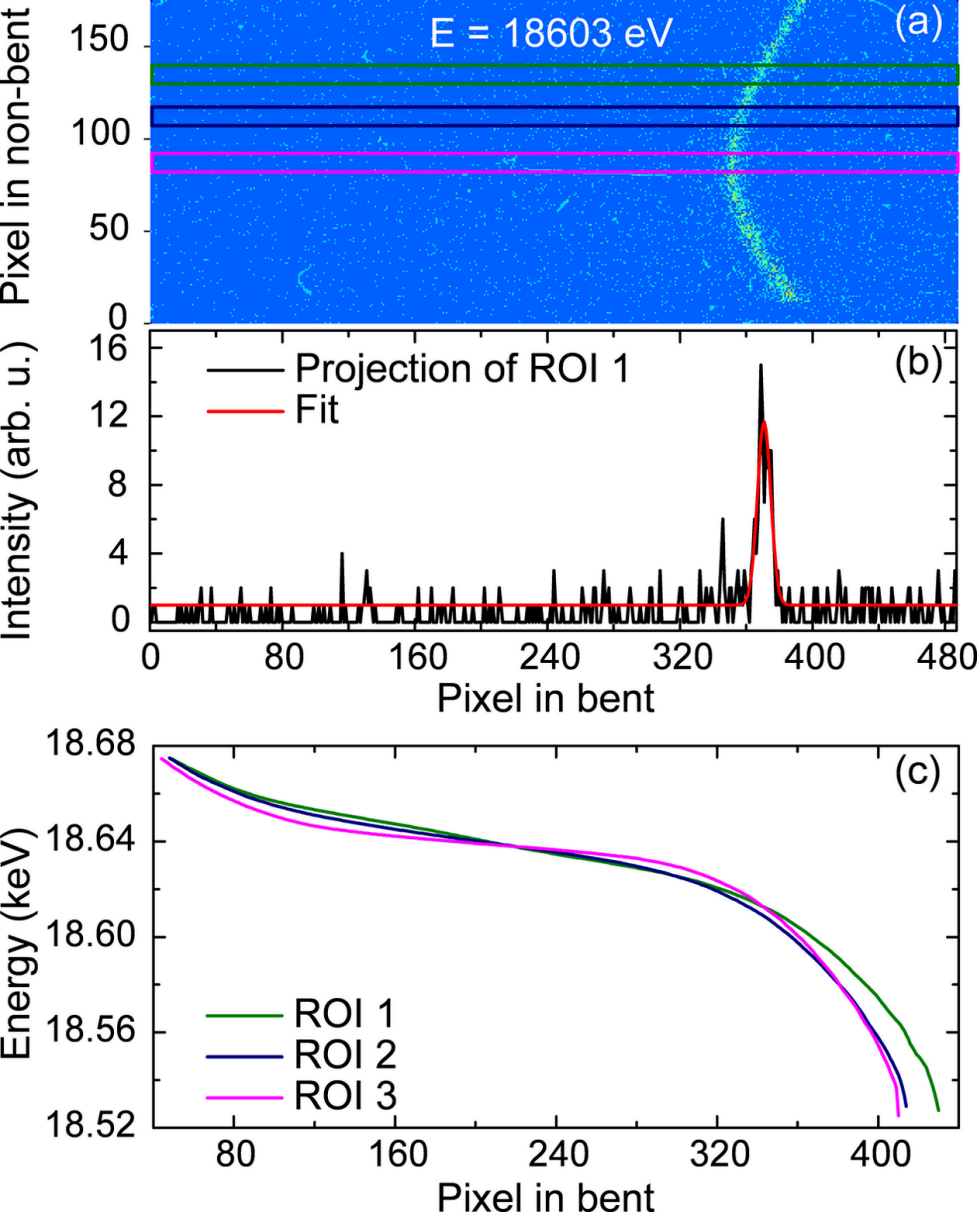
Conceptual steps of the slicing algorithm for energy calibration. Panel (*a*) shows an example elastic scattering image. The rectangular bars represent the ROIs used in the projection and fitting. A Gaussian fitting is employed to extract the peak position of the pixels (*b*), ROI 1 is used as an example of projection. In batch fitting, the initial parameters of peak position are prepared by using a peak-finding function from the filtered image. This allows the ROI to be selected as narrowly as possible, despite the pronounced noise level. (*c*) Representative pixel–energy relation for different ROIs along the non-bent axis. The colors correspond to the ROIs used in panel (*a*).

An alternative method for calibrating the energy axis is a modified approach of projection along the non-dispersion axis. If the analyzer is properly aligned, the energy dispersion in the bent direction can exhibit monotonic behavior, and each narrow vertical region on the analyzer can be approximated as a straight line. Thus, each of these vertical slices can be projected creating a pixel-to-energy mapping for the different vertical regions. The narrowest slice possible, with a height of only one pixel, is ideal for this procedure. However, using a wider slicing region and summing the intensities inside it was necessary to increase the elastic signal intensity. Slices with a height of ten pixels were used in the energy calibration. An illustration of such slices taken on the emission image corresponding to 18603 eV is shown in Fig. 10 ▸(*a*), together with the associated projection and Gaussian fit to the elastic peak in Fig. 10 ▸(*b*). A batch of slicing and fitting was applied to all elastic scattering images corresponding to the different energies and the pixel-to-energy correspondences were obtained for every slice. Subsequently, an interpolation in the per pixel step was applied for each slice in order to account for the different dispersion along the non-bent direction. Examples of these pixel-to-energy calibration curves for three different vertical slices are shown in Fig. 10 ▸(*c*).

The pixel-to-energy correspondence is clearly non-linear, with notable differences across the different slice regions along the non-bent direction. This results in non-uniform energy steps across the spectrum, which can be corrected by re-binning. After re-binning, the emission spectra from different slices are summed to generate the final XES spectrum. In this method, the energy sampling only depends on the detector pixel size and avoids the need to collect a large elastic scattering data set with small energy intervals. The final energy calibration yields a relation of ∼0.5–1.0 eV per pixel across the emission spectrum.

Fig. 9 ▸ shows the *K*β_1_ and *K*β_3_ XES from a niobium foil using the slicing algorithm (solid line). This spectrum was obtained from the same emission image used in the mask algorithm (dots). The XES spectra obtained using both algorithms are comparable, demonstrating the robustness of both methods of image processing. Their good agreement suggests that the emission energy in each image could also be calibrated using a set of referenced XES spectra, thus avoiding the need to collect monochromatic elastic scattering data.

### 4.2. Energy resolution and efficiency at SuperXAS

**Figure 11:**
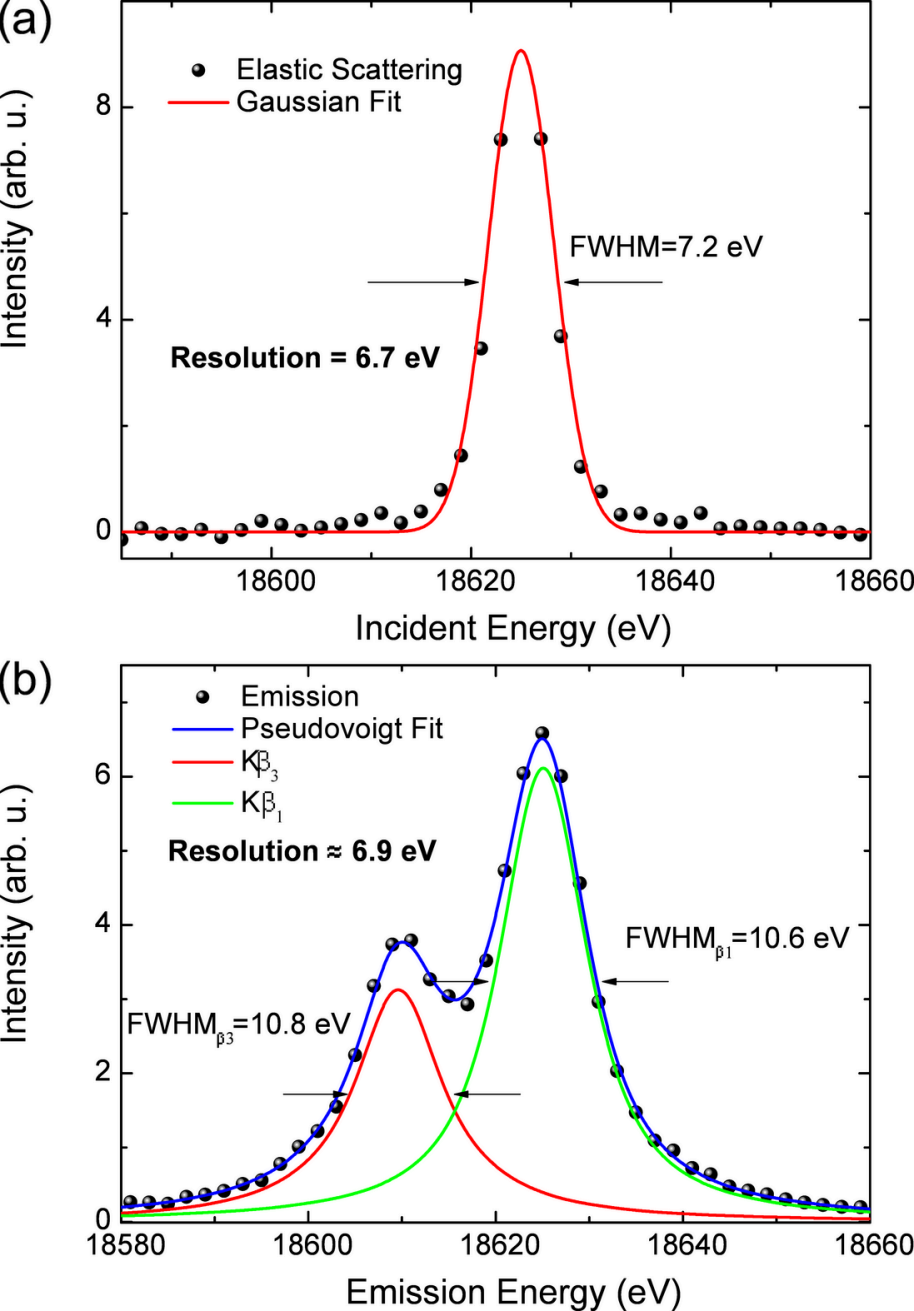
Evaluation of energy resolution for the Laue analyzer using experimental data from the mask algorithm. (*a*) The elastic scattering of Nb foil, the incident energy is at 18625 eV, the intrinsic energy resolution of the silicon (111) monochromator is 2.6 eV. The resolution of the Laue spectrometer is obtained by deconvolving the two Gaussian functions. (*b*) The *K*β_1,3_ emission spectra of the Nb foil, the resolution of the Laue spectrometer is obtained by deconvolving the Lorentzian and Gaussian functions.

The Laue spectrometer energy resolution was evaluated using the elastic scattering data as well as by deconvoluting the peak width of the *K*β_1,3_ emission line. The approaches resulted in comparable results. A representative measurement of the elastic scattering at 18625 eV is shown in Fig. 11 ▸(*a*) together with a fit using a Gaussian profile. The fitted FWHM of this Gaussian curve was 7.2 eV. Considering the intrinsic energy resolution of Si(111) monochromators at this energy (about 1.4 × 10^−4^) is on the order of 2.6 eV, a deconvolved spectrometer resolution of 6.7 eV is obtained. This value is slightly worse than the 5.18 eV obtained in the work of Jagodzinski *et al.*, likely due to the longer analyzer radius (2 m) used in their setup (Jagodziński *et al.*, 2019 ▸). Fig. 11 ▸(*b*) presents the *K*β_1,3_ XES of a Nb foil and the fitted pseudo-Voigt functions used in the deconvolution. The instrument response is described by the Gaussian component and the Lorentzian one accounts for the 6.2 eV of the natural linewidth of the Nb *K*β_1_ emission (Campbell & Papp, 2001 ▸). Considering these, the final spectrometer resolution is about 6.9 eV. The slight difference of about 3% in the resolution obtained using the two evaluation methods is attributed to inaccuracies in the monochromatic bandwidth and the theoretical Nb *K*β natural linewidth. This indicates that collecting a complete elastic scattering data set is not strictly necessary to evaluate the spectrometer energy resolution. The energy resolution was limited mainly by contributions due to the horizontal beam size (∼140 µm, already taking into account the projection footprint on the sample at ∼45°) used at SuperXAS. The measured energy resolution is comparable with the calculated value using X-ray tracing as shown in Fig. 4 ▸(*c*). This excludes significant effects of analyzer surface distortion, image processing and detector pixel size on the Laue spectrometer energy resolution. A better energy resolution could be expected at FXE and other beamlines when using smaller beam sizes (∼20 µm). In this limit, using detectors with a smaller pixel size can also result in improved energy resolution.

**Figure 12:**
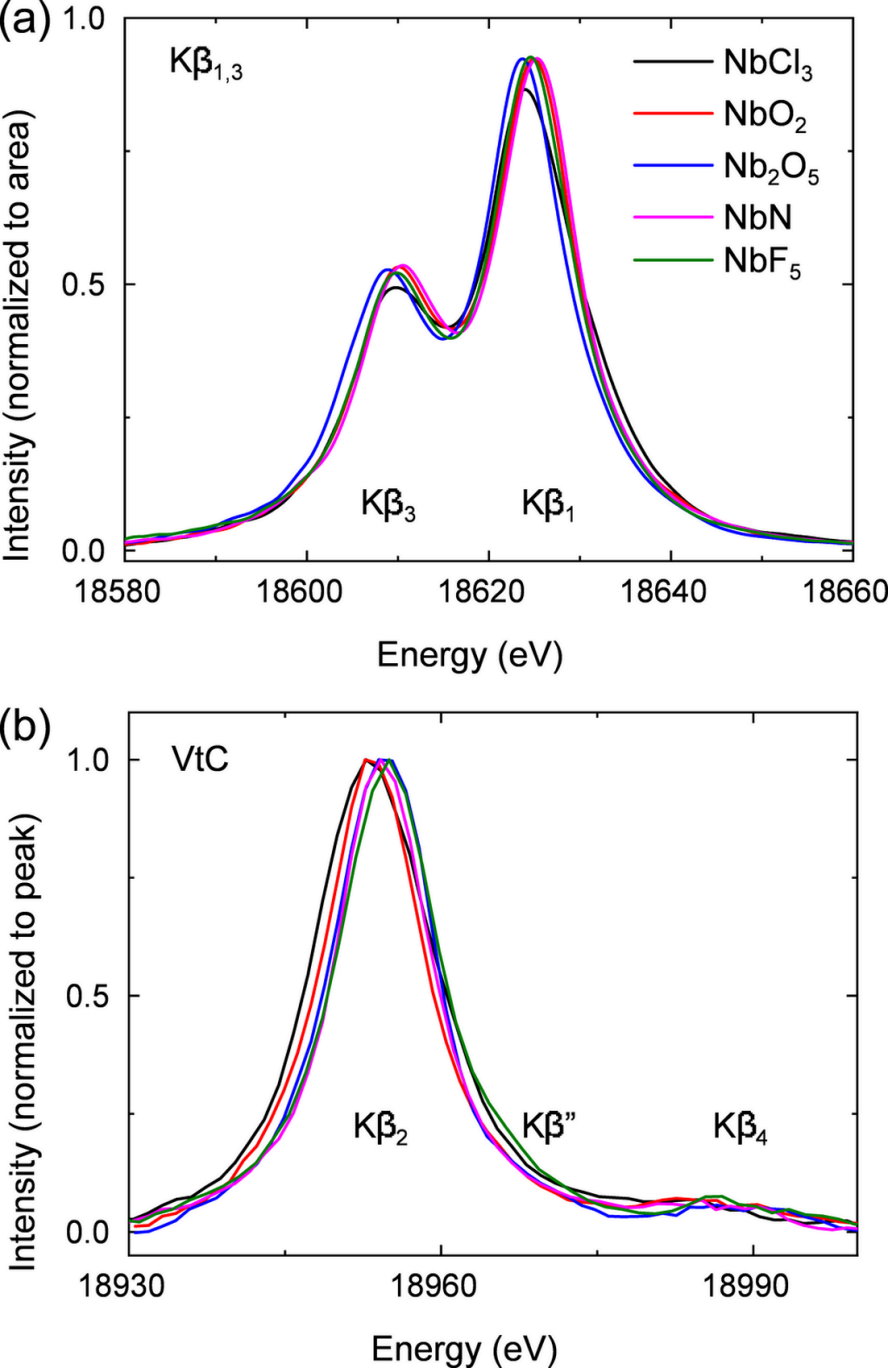
XES spectra of different niobium compounds with different electronic states: Nb^III^Cl_3_, Nb^IV^O_2_, Nb
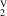
O_5_, Nb^V^N and Nb^V^F_5_. All samples were purchased from Sigma–Aldrich as powders, which were mixed with boron nitride and pressed into pellets. The signal background from all spectra was removed. Panel (*a*) shows the *K*β_1,3_ spectra and the peak profiles were normalized to the area (to better show the profile difference); panel (*b*) shows the valence-to-core (VtC) spectra, also normalized to the peak intensity (to better show the peak position difference).

We have used the Laue spectrometer to measure the *K*β_1,3_ XES of a series of solid niobium compounds with varied oxidation state and ligand environment. The spectra of these reference compounds are shown in Fig. 12 ▸. The dispersed image of the Nb *K*β_1,3_ emission was clearly visible on the detector in less than 100 s (∼10^11^ photons s^−1^ incoming flux at SuperXAS), contrasting with around 1 h in total (45 s per point in scanning mode) used to measure the Ru *K*β_1,3_ at the same beamline with the spectrometer described by Jagodzinski *et al.* We have also measured the weaker VtC emission of the same Nb reference [see Fig. 12 ▸(*b*)]. Each measurement detecting the *K*β_2_, *K*β′′ and *K*β_4_ lines could be completed in ∼30 min, while similar data collection on ruthenium oxides took 12 min per data point, resulting in about 10 h of total measurement. As shown in Table 1 ▸, the separations between the peaks of *K*β_2_ and VtC features of Nb_2_O_5_ and NbF_5_ samples agree well with the values reported by Ravel *et al.* (2018 ▸). Importantly, the subtle *K*β′′ and *K*β_4_ on the Ru references were not clearly visible in the measurements using the previous Laue spectrometer from SuperXAS.

In terms of efficiency, our spectrometer outperforms the previous implementation at SuperXAS using a dynamically bent Laue analyzer. The analyzer optimization, in particular the choice of thinner crystals and an asymmetric cut, combined with a new design of the holding frame resulted in an increased spectrometer efficiency without a significant impact on the energy resolution. Moreover, operation in dispersive mode was successfully demonstrated. These results are encouraging, when considering the implementation of high-resolution ultrafast pump–probe spectroscopies at high X-ray photon energies at XFELs using Laue spectrometers.

## 5. HELIOS implementation at FXE

After the tests at a synchrotron beamline, the Laue spectrometer was fully assembled, installed and commissioned at the FXE instrument. Two Laue analyzers, one silicon and one quartz, were tested profiting from the strong pulse intensities at high photon energies provided by the European XFEL operation at 16.3 GeV. The Laue spectrometer as discussed in Section 3 was designed to be docked into the SMS [see Fig. 6 ▸(*b*)]. This allows the scattering angle to be varied in future experiments exploiting momentum-transfer dependence. In the measurements discussed here the spectrometer was placed perpendicular to the beam propagation direction where the elastic and Compton scattering intensities are weakest. This geometry allowed simultaneous operation of the Laue and von Hamos spectrometers at FXE as shown in Fig. 6 ▸(*b*), providing an opportunity for a direct comparison of signal levels, efficiency and resolution of Laue and Bragg analyzers within a single measurement.

Briefly, the XES measurements at FXE using the Laue spectrometer were done using a SASE (self-amplified spontaneous emission) beam of 19.2 keV photon energy with a pulse intensity of ∼550 µJ, as measured by an X-ray gas monitor detector installed in the XFEL tunnel. The X-ray beam was focused down to ∼20 µm × 20 µm using beryllium lenses. Niobium *K*α_1_ and *K*β_1,3_ XES spectra of a metallic foil and different oxides (NbO_2_ and Nb_2_O_5_) were analyzed using the third-order reflection of Si(111) and SiO_2_(110) Laue crystals. All samples were mounted in the center of the SMS at a distance of about 1.4 m from the Laue analyzer. The Bragg angle for measuring Nb *K*α_1_ was 20.9° and 27.1° when using Si(333) and SiO_2_(330), respectively, and for measuring *K*β_1,3_ it was 18.6° and 24.0° when using Si(333) and SiO_2_(330), respectively. Initially, the strong Nb *K*α_1_ emission line was used to calibrate the spectrometer motor stages and optimize the signal levels. The Laue spectrometer was set for dispersive operation with the analyzer placed around −8 mm out of the Rowland circle. Possible damage to the solid samples induced by the intense focused XFEL beam was avoided by attenuating the incoming beam to ∼40% total transmission during *K*α data collection and ∼20% for *K*β. The combined beamline transmission including the focusing lenses, attenuators, beam imaging screens and windows was estimated to be 10%–20%. A total of 10–20 pulses per train at a 282 kHz intra-train repetition rate (100–200 pulses per second) were used during the measurements, resulting in an incoming flux comparable with that of SuperXAS. In regular operation conditions up to around 200 pulses per train at 0.5 MHz (equivalent to 2000 pulses per second) can be used at FXE. A total of eight Si(111) cylindrical analyzers with 0.5 m radius were installed in the von Hamos spectrometer and placed at about 73° to analyze the Nb *K*α emission. Interestingly, the Bragg angles for Nb *K*α_1_ (16.61 keV, 72.16°), *K*α_2_ (16.52 keV, 73.21°) and *K*β (18.62 keV, 72.84°) when using Si(111) crystals are very similar, with the eighth diffraction order used for the former and ninth used for the latter. One cylindrical crystal used in the von Hamos spectrometer has a size of 11 cm × 3 cm, which is comparable with the Laue crystal. The same detector type, namely the JUNGFRAU, was used on the von Hamos (JF-1M) and Laue (JF-500 K) spectrometers. Benefiting from the modest energy resolution of the JF detector (1–2 keV), a lower signal threshold was set to remove the electronic noise (<2.5 ADU) and secondary scattering (*e.g.* background fluorescence from the lead shielding around the detector, ∼13 ADU) during the image processing, which helped to improve the signal-to-background ratio.

### 5.1. Energy calibration at XFELs: self-calibration method

The use of elastic scattering to calibrate the energy axis on measurements using the Laue analyzer at XFELs is not convenient because of the following limitations: (i) the Laue spectrometer is installed perpendicular to the beam propagation direction where elastic scattering is weakest, and (ii) elastic scattering measurements require the monochromator to be tuned to an energy close to that of the analyzed fluorescence line, which in the case of Nb *K*α implies a change of more than ∼2.4 keV. Such large energy changes on XFEL undulators operating in SASE mode are not straightforward. Additionally, the use of a monochromator can limit the total number of X-ray pulses in each burst train at the European XFEL, translating into a lower overall output. Combined, these factors make the collection of elastic scattering data sets at XFELs significantly more cumbersome and time-consuming when compared with synchrotrons.

**Figure 13:**
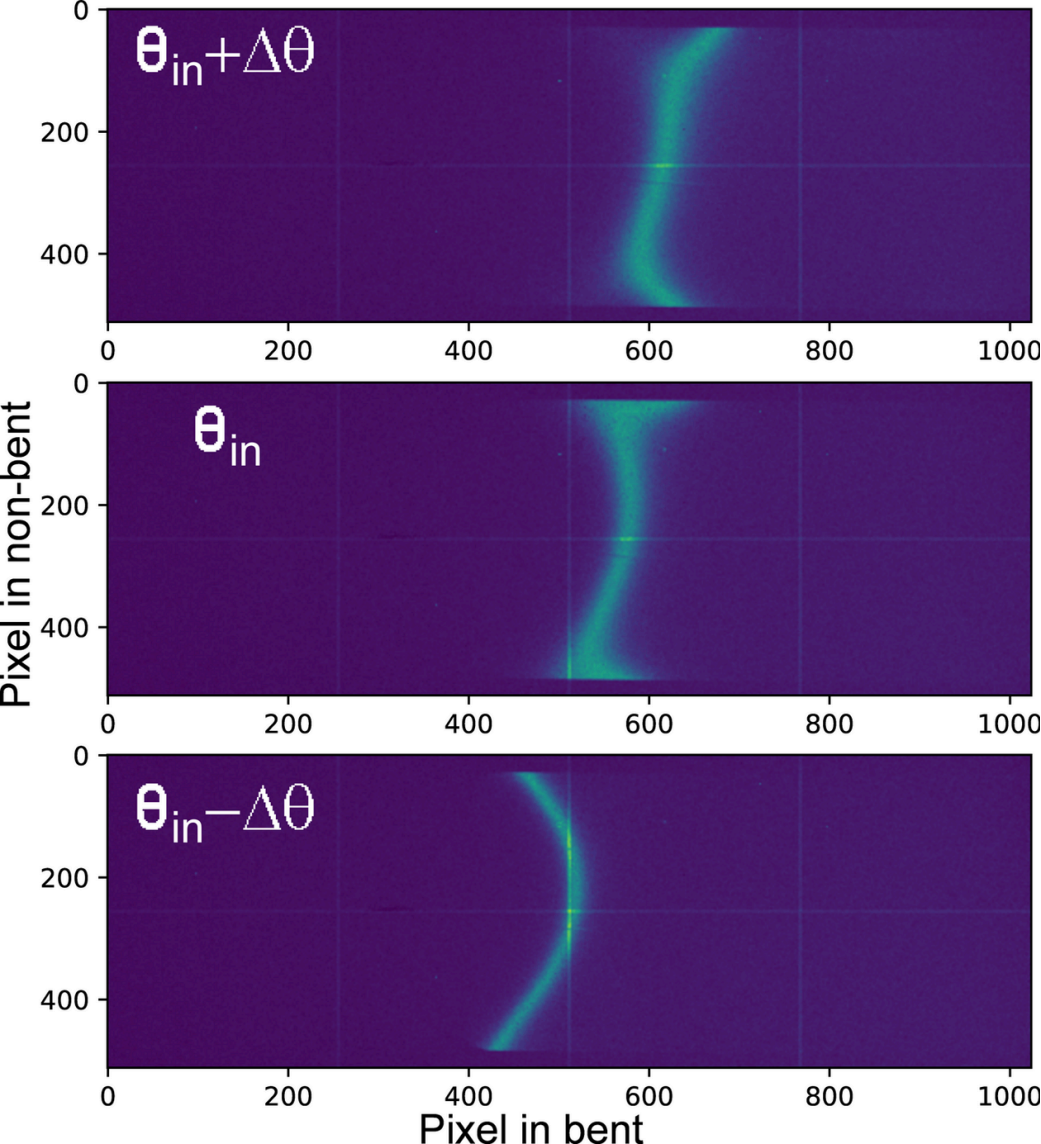
Example emission stripes collected at different incident angles. The stripe moves from the right side to the left side as the incident angle decreases. The signal is from a metallic Nb foil.

An alternative approach to the energy calibration is proposed here based on the basic concept of dispersive optics. The dispersion property of Laue analyzers arises from the fact that different energies originating at the source have different incident angular offsets along the analyzer bent axis. This is illustrated in the scheme of Fig. 3 ▸(*b*). This behavior is equivalent to changing the angular offset by rotating the analyzer while keeping the detector position fixed when a single photon energy is emitted from the source. Fig. 13 ▸ illustrates this effect in the measurement of Nb *K*α_1_ emission collected at different angles. The signal, in the form of a vertical stripe, moves from right to left along the bent axis with decreasing incident angle on the analyzer, exhibiting a behavior similar to that observed during an energy scan in elastic scattering [Fig. 8 ▸(*b*)]. Similar to the slicing and re-binning algorithm discussed in Section 4, a 2D calibration map using angles instead of energies can be prepared and used for the energy calibration. For any given emission feature, *e.g.* the single peak of *K*α_1_ or double peaks of *K*β_1,3_, the pixel coordinate of each peak position can be extracted by slicing the image along the non-bent direction and batch fitting. The emission image is then re-binned into a spectrum and the angles can be converted into energies using the Bragg equation and compared with a reference emission spectrum. This method is referred to as self-calibration.

**Figure 14:**
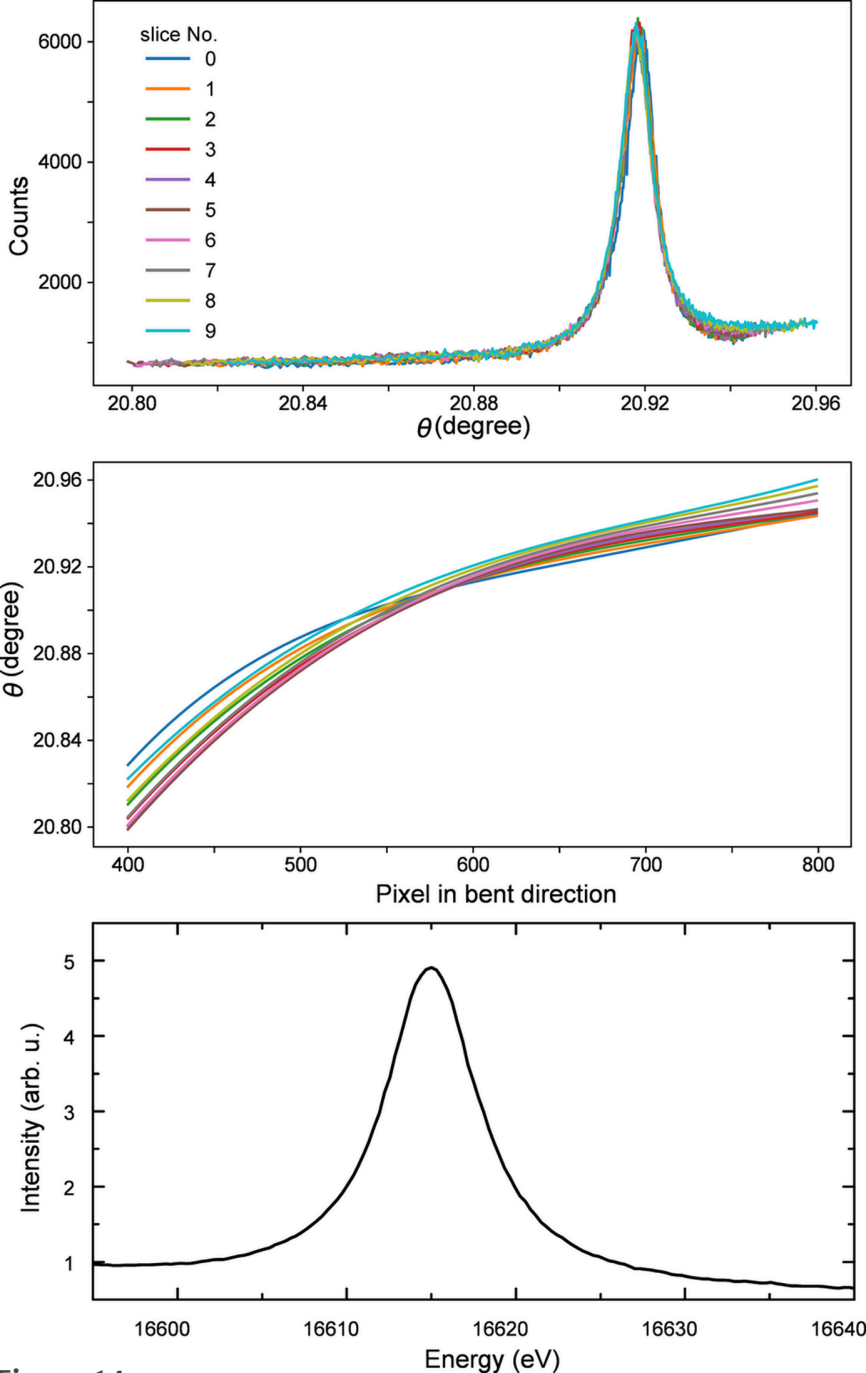
The basic concept to convert the emission image into a spectrum by using the self-calibration method. Top panel is the sliced spectra after pixel calibration based on the angular scan showing good overlap of the peak position. Middle panel is the pixel–angle calibration at different slice regions; non-linear and non-uniform relations are shown. Bottom panel is the emission spectrum of Nb *K*α_1_ after re-binning and transforming angles to energies. The signal is from Nb foil sample.

Fig. 14 ▸ illustrates the steps necessary to convert the emission images to a spectrum using the self-calibration method. The data were collected using a 250 µm-thick Nb metallic foil as sample. Slices with a height of 30 pixels along the non-bent axis were sufficient to fulfill the assumption that within that small ROI, the emission stripe can be regarded as a straight line while providing sufficient intensity for an accurate determination of the peak positions. A similar batch fitting as used for the slicing algorithm (illustrated in Fig. 10 ▸) is then applied to extract the angle-to-pixel calibration relation for all slices within a given emission image. As expected, a non-linear and non-uniform relation between angle and pixel is also observed. The results of these procedures are summarized in the top and middle panels of Fig. 14 ▸. Finally, all spectra are re-binned and summed to form the emission spectrum as a function of angle. The last step is to identify the peak angular position and convert it into energy using the Bragg equation and the reference energy value for Nb *K*α_1_ (16615 eV). The inter-planar *d*-spacing values from the silicon (111) and quartz (110) analyzers were taken from the *XOP* package (Sanchez del Rio & Dejus, 2011 ▸). Similar values were reported by the analyzer manufacturer (Saint-Gobain). The energy step per pixel varied from 0.2 eV to 0.5 eV, depending on the different dispersive regions along the non-bent direction. One additional effect to consider is the non-uniform background. Specifically, the low-energy region (larger angles) has a larger background than the high-energy one. This is due to the geometry used: photons scattered by the sample and transmitted through the Laue analyzer and its supporting frame contribute more to the background for the higher angular region than for the lower angular region. This effect could be minimized by the use of a trapezoidal shaped flight path between the sample and Laue analyzer [see Fig. 6 ▸ (*b*)] and additional shielding around the detector and along the beam path before and after the sample.

**Figure 15:**
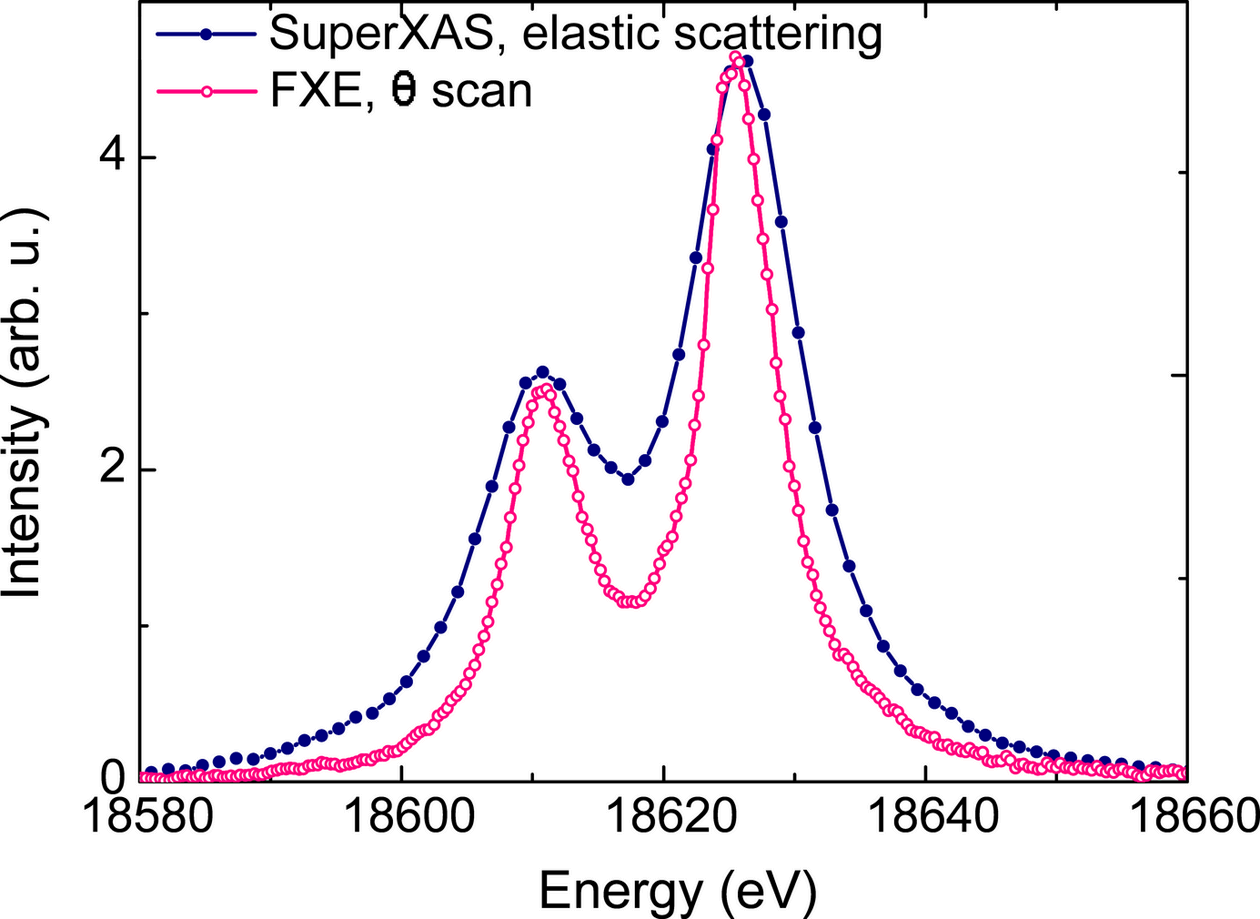
Comparison of the *K*β_1,3_ emission spectrum of Nb foil collected at SuperXAS and FXE. The background for each spectrum was subtracted for better comparison. The SuperXAS spectrum is calibrated by the elastic scattering scan and the FXE data are calibrated by the angular scan in the self-calibration method.

The accuracy of the self-calibration method was evaluated by comparing the Nb *K*β_1,3_ emission spectrum collected at FXE with that collected at SuperXAS, which was calibrated using elastic scattering. The result of this comparison is shown in Fig. 15 ▸. In the FXE data, the energy is converted from angle using only one energy position of *K*β_1_ peak position. The absolute energy of the *K*β_1_ and *K*β_3_ peaks and their energy separation are similar in both data sets. A slight difference of around 3% in the energy separation was observed, likely due to inaccuracies in the elastic scan calibration method and worse energy resolution in the synchrotron data set. Importantly, this difference is smaller than the Si(111) monochromator bandwidth used in the elastic scan calibration. The data can be further re-scaled and corrected by using additional energy positions on the reference spectrum. Moreover, there is a large difference in the peak widths due to the better energy resolution of the FXE data.

### 5.2. Energy resolution and efficiency at FXE

The comparison between the two Nb *K*β XES spectra using the same Si(111) analyzer shown in Fig. 15 ▸ shows the improved energy resolution obtained in the measurements at FXE. The main factors contributing to a better energy resolution are the smaller beam size of 20 µm (compared with 100 µm at SuperXAS) and the smaller detector pixel size (75 µm for the JUNGFRAU at FXE versus 172 µm for the Pilatus at SuperXAS), combined with setting the dispersion on the vertical axis (implying the beam footprint on the sample at 45° does not affect the beam size in the dispersion direction).

**Figure 16:**
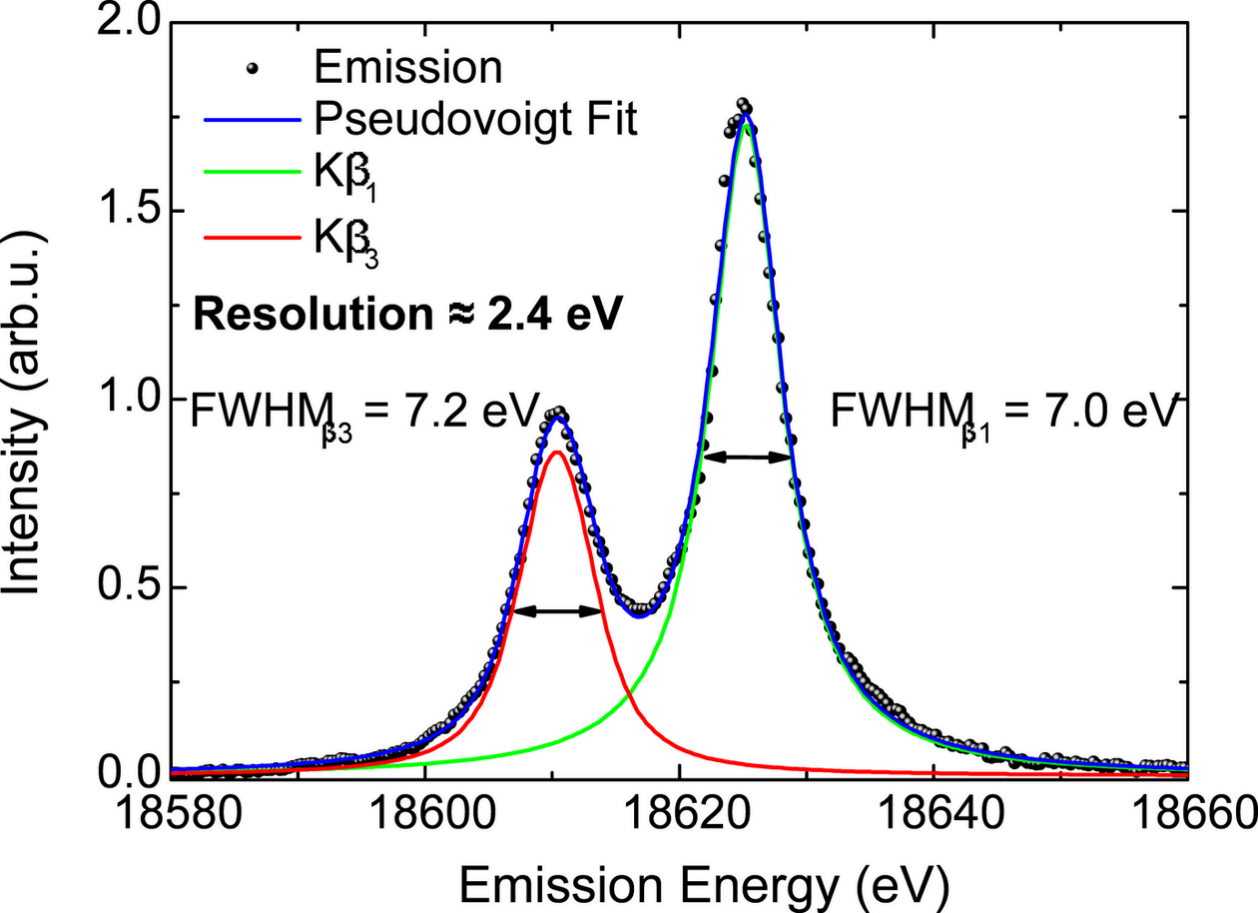
Evaluation of energy resolution of the Laue spectrometer using a Si(333) analyzer. The *K*β_1,3_ emission spectrum is collected from the Nb foil. The resolution of the Laue spectrometer is obtained by deconvolving the Lorentzian and Gaussian functions. A resolution of 2.4 eV is obtained by using the Lorentzian function to account for the natural linewidth of 6.2 eV, as reported by Campbell & Papp (2001 ▸).

Given the absence of elastic scattering data on the XES measurements collected at FXE, the energy resolution was evaluated by directly deconvoluting the *K*β emission spectrum using the same procedure employed in the SuperXAS data. The Nb *K*β XES spectrum measured using the Si(111) Laue analyzer in the third diffraction order was fitted using two pseudo-Voigt functions describing the *K*β_1_ and *K*β_3_ peaks and the results are shown in Fig. 16 ▸. The instrument response is deconvoluted from the fit considering a Lorentzian function with a natural width of either 6.2 eV according to the calculated value reported by Campbell & Papp (2001 ▸) or 6.4 eV obtained from the measurement in Fig. 11 ▸. These resulted in a spectrometer resolution of 2.4 eV or 2.1 eV, depending on the value used for the Nb *K*β linewidth. That corresponds to a Δ*E*/*E* ≃ 1.1 × 10^−4^ to 1.3 × 10^−4^, which is approximately three times better than the value obtained at SuperXAS.

**Figure 17:**
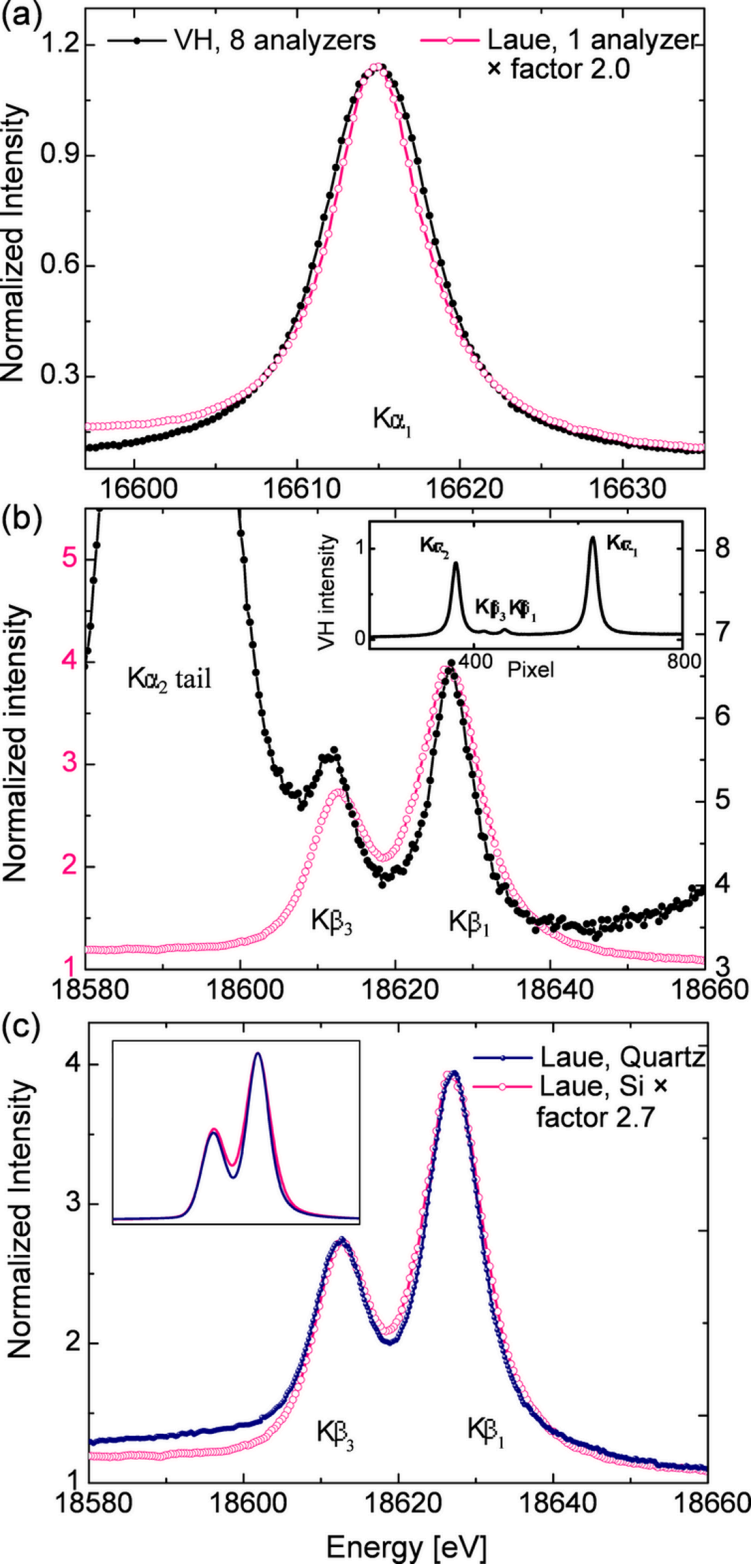
Performance comparisons between the von Hamos spectrometer with eight Si(111) Bragg crystals and the single Si(333) Laue crystal and between the Si(333) and SiO_2_(330) analyzers. (*a*) *K*α_1_, and (*b*) and (*c*) *K*β_1,3_ spectra from a Nb foil. The background in each data set was kept here to provide a realistic performance comparison, and the background-subtracted spectra are shown in the inset of (*c*). To carefully compare the efficiency at the *K*β energy, a larger beam size (∼40 µm), *i.e.* worse energy resolution for the Laue analyzer, was used to prevent sample damage, as acquiring the *K*β signal takes longer compared with the *K*α signal. The inset of (*b*) shows the full spectra measured using the von Hamos spectrometer, where *K*α and *K*β lines are collected by (888) and (999) reflections, respectively.

Fig. 17 ▸ presents niobium XES comparing different performance aspects of these measurements: in panel (*a*) a comparison of the *K*α_1_ emission collected using the von Hamos and Laue analyzers is shown, in panel (*b*) the *K*β emission measured using von Hamos and Laue analyzers is compared, and in panel (*c*) the *K*β emission measured using silicon and quartz Laue crystals is compared. Using the (330) reflection of a quartz Laue analyzer with similar radius for measuring the same Nb *K*β XES resulted in a spectrum with slightly better resolution compared with the data collected using the silicon analyzer. The comparison of the two data sets is shown in Fig. 17 ▸(*c*). Further improvements in energy resolution can be achieved by using higher diffraction orders; however, the narrower Darwin width associated with high diffraction orders leads to lower integrated signal efficiency. An improvement factor of around 1.7 is predicted from the X-ray tracing when using Si(555) instead of Si(333), while giving about a factor 1.9 lower efficiency.

As stated before, the von Hamos spectrometer was equipped with eight Si(111) cylindrical analyzers, each with a similar size to the Laue analyzers. The emission signals collected with either spectrometer were normalized to the incoming flux, acquisition time and the absorption by the air path (distance from sample to analyzer). The background on each spectrum was retained in order to provide a realistic comparison of the spectrometer performance. In terms of efficiency, one silicon Laue analyzer with 1.5 m radius is comparable with four silicon cylindrical von Hamos analyzers with 0.5 m radius at the Nb *K*α energy (∼16.6 keV), and comparable with eight von Hamos analyzers at the *K*β energy (∼18.6 keV). It can be observed that the efficiency of the Bragg crystal gradually decreases with increasing energy with respect to that of the Laue crystal. It is important to note the measurements with both spectrometers shown in Figs. 17 ▸(*a*) and 17 ▸(*b*) were collected simultaneously. Comparing the spectra measured with the two different Laue analyzers indicated a ∼2.7 times better efficiency when using the quartz analyzer instead of the silicon, which agrees with the rocking-curve calculations shown in Section 2.2. The Laue analyzer manufacturing technology is progressing in the direction of reducing the bending radius to below 1 m, which should result in an additional twofold increase in efficiency. The results shown here demonstrate the superior efficiency of the Laue analyzer with respect to Bragg crystals at energies >15 keV. At even higher photon energies, *e.g.* when measuring *K*α or *K*β emission lines of Pd or Ag (∼21.1–24.9 keV), the improved efficiency of Laue analyzers will become even more pronounced.

Regarding energy resolution, using the Laue analyzers resulted in spectra with resolution comparable with that measured with the von Hamos spectrometer. The pixel-to-energy positions in the von Hamos data were calibrated using two peak positions of either *K*α_1, 2_ lines [Fig. 17 ▸(*a*)] or *K*β_1,3_ lines [Fig. 17 ▸(*b*)] assuming a linear dispersion. The energy resolution in the von Hamos data is worse than expected without considering the much larger dispersion window and other effects due to the larger penetration of the harder X-rays into the analyzer crystals before being diffracted, *e.g.* strain in the crystal planes, deformations in the crystal–substrate interface *etc*. The spectra measured with the Laue quartz analyzer have slightly better energy resolution than those using the Laue Si one, being 2.2 eV and 2.4 eV, respectively. This difference is likely due to the different Bragg angles needed for the same measurement using different analyzers – for SiO_2_(330) a larger angle is needed than for Si(333). At the Nb *K*β_1_ energy the angles are 24.0° and 18.6°, respectively. The comparisons between the von Hamos and Laue spectrometers are summarized in Table 2 ▸.

## 6. Conclusions and future perspectives

We have presented the design and first commissioning results of a new X-ray spectrometer for high X-ray photon energies using Laue analyzers. The High-Energy Laue X-ray emIssiOn Spectrometer installed at the FXE instrument at the European XFEL was optimized for improved efficiency and ease of operation, while maintaining a total energy resolution below the core-hole lifetime of 4*d* elements. This spectrometer significantly increases the X-ray spectroscopic capabilities of FXE by enabling unique high-resolution X-ray spectroscopy experiments at high photon energies. To the best of our knowledge, this is the only spectrometer of this kind at any XFEL facility worldwide.

Two Laue analyzers, a silicon (333) and a quartz (330), with bending radius of ∼1.5 m were used in the measurements. A silicon analyzer was initially tested in dispersive mode at the SuperXAS beamline of the Swiss Light Source, providing an energy resolution of 3.6 × 10^−4^. This limitation was mainly due to the relatively large beam and detector pixel sizes at SuperXAS. An improved energy resolution of ∼1.2 × 10^−4^ was obtained in the measurements at FXE using a quartz analyzer. The smaller beam (20 µm) and detector pixel sizes (75 µm) were the main factors contributing to this improvement. The energy dispersion capabilities of our Laue spectrometer were shown to be a convenient and effective way of measuring XES spectra at high photon energies. Our Laue spectrometer has demonstrated the ability to resolve the *K* emission spectra of 4*d* metals at high X-ray energies, detecting an energy interval of ∼100 eV, sufficient for covering a large portion of the *K*β emission at once.

Several image processing methods have been developed to convert the emission images to spectra. Distortions in the emission images were observed due to the nature of Laue crystal surfaces and the defocused geometry. Despite this, effective algorithms for data reduction have been developed for reconstructing the spectra. Using either elastic scattering data sets or analyzer angular scans allows for accurate corrections and conversions of the emission images to XES spectra in a robust manner. In particular, the self-calibration method using angular scans can also be used to correctly calibrate the spectrometer, thereby circumventing the need for elastic scattering measurements and addressing operational inconveniences of XFELs operating in SASE mode.

The Laue spectrometer reported here has demonstrated a significant improvement in efficiency when compared with the (Bragg) von Hamos spectrometer at high photon energies. This was shown by simultaneous measurements using both spectrometers, which indicated an increase of a factor of ∼4–22 in the signal strength measured with the Laue spectrometer, depending on the analyzer material, emission line and number of analyzers used. In fact, similar conclusions regarding the higher efficiency of Laue analyzers at high photon energies have been recently reported by Hiraoka (2025 ▸). The first Nb *K*β XES results collected at SuperXAS also indicate a significant efficiency improvement compared with their previous spectrometer equipped with a dynamic bent analyzer with larger radius and operated in scanning mode. Clear features in VtC and *K*β regions of the XES spectra could be measured in about half an hour and just several minutes, respectively. Moreover, the HELIOS spectrometer is equipped with a thin analyzer frame and a large aperture, which combined with motorization of all analyzer and detector degrees of freedom offer the ability to choose different diffraction planes without shadowing the detector. This allows the measurements to be tuned for a compromise between energy resolution and efficiency, depending on different measurement scenarios. For instance, valence-to-core emission spectra can be measured using modest resolution with lower diffraction orders for higher efficiency, while *K*β lines, X-ray Raman scattering and other emission lines requiring higher energy resolution can benefit from a better resolution scheme. By utilizing the high X-ray energy capabilities and the improved energy resolution and efficiency of the Laue spectrometer, FXE will enable several novel spectroscopic capabilities, *e.g.* non-resonant and resonant XES, and HERFD-XAS at high photon energies. These advancements will inspire new ultrafast pump–probe spectroscopic studies in the 15–20 keV range, covering the *K* lines of 4*d* elements and *L* lines of 5*f* elements.

## Figures and Tables

**Table 1:** Fitted peak positions of VtC features of the niobium samples (uncertainties are given as standard deviations of each individual fit)

Sample	*K*β_2_ (eV)	*K*β*′′* (eV)	*K*β_4_ (eV)
NbCl_3_	18954.3 (± 0.1)	18964.5 (± 3.6)	18984.2 (± 1.1)
NbO_2_	18953.6 (± 0.2)	18966.3 (± 2.2)	18988.2 (± 3.5)
Nb_2_O_5_	18954.7 (± 0.1)	18969.9 (± 2.2)	18987.2 (± 1.2)
NbN	18955.2 (± 0.3)	18967.9 (± 2.7)	18983.5 (± 3.6)
NbF_5_	18955.3 (± 0.2)	18968.2 (± 1.7)	18987.0 (± 1.8)

**Table 2:** Comparison between the von Hamos (VH) and Laue spectrometers The efficiency values of the von Hamos spectrometer in *K*α and *K*β measurements are set to 1.

	VH	Laue-Si	Laue-SiO_2_
Crystal	Silicon	Silicon	Quartz
Radius (m)	0.5	1.5	1.5
Open window	3 cm × 11 cm	3 cm × 8 cm	3 cm × 8 cm
Index	(111)	(111)	(110)
Efficiency at Nb *K*α	1	∼4	∼11
Efficiency at Nb *K*β	1	∼8	∼22
Resolution at Nb *K*β (eV)	1.9	2.4	2.2
